# Polyphenols: Extraction Methods, Antioxidative Action, Bioavailability and Anticarcinogenic Effects

**DOI:** 10.3390/molecules21070901

**Published:** 2016-07-11

**Authors:** Eva Brglez Mojzer, Maša Knez Hrnčič, Mojca Škerget, Željko Knez, Urban Bren

**Affiliations:** 1 Laboratory of Physical Chemistry and Chemical Thermodynamics, Faculty of Chemistry and Chemical Engineering, University of Maribor, Smetanova ulica 17, SI-2000 Maribor, Slovenia; eva.spaninger@um.si; 2 Laboratory of Separation Processes and Product Design, Faculty of Chemistry and Chemical Engineering, University of Maribor, Smetanova ulica 17, SI-2000 Maribor, Slovenia; masa.knez@um.si (M.K.H.); mojca.skerget@um.si (M.Š.)

**Keywords:** polyphenols, extraction, antioxidants, cancer, bioavailability, synergistic effects

## Abstract

Being secondary plant metabolites, polyphenols represent a large and diverse group of substances abundantly present in a majority of fruits, herbs and vegetables. The current contribution is focused on their bioavailability, antioxidative and anticarcinogenic properties. An overview of extraction methods is also given, with supercritical fluid extraction highlighted as a promising eco-friendly alternative providing exceptional separation and protection from degradation of unstable polyphenols. The protective role of polyphenols against reactive oxygen and nitrogen species, UV light, plant pathogens, parasites and predators results in several beneficial biological activities giving rise to prophylaxis or possibly even to a cure for several prevailing human diseases, especially various cancer types. Omnipresence, specificity of the response and the absence of or low toxicity are crucial advantages of polyphenols as anticancer agents. The main problem represents their low bioavailability and rapid metabolism. One of the promising solutions lies in nanoformulation of polyphenols that prevents their degradation and thus enables significantly higher concentrations to reach the target cells. Another, more practiced, solution is the use of mixtures of various polyphenols that bring synergistic effects, resulting in lowering of the required therapeutic dose and in multitargeted action. The combination of polyphenols with existing drugs and therapies also shows promising results and significantly reduces their toxicity.

## 1. Introduction

Nowadays nutrition is gaining importance as the basic foods are processed in a way whereby organoleptic properties prevail over their nutrient composition. That is the main reason behind the widespread obesity problem and also for the appearance of several currently prevailing diseases.

Studies have shown that correct dietary habits, including consumption of plenty and various fruits, legumes and grains can prevent 10%–70% of cancer deaths [1]. However, the substances in listed foods that mainly contribute to disease prevention and healing have only been discovered not so long ago [2]. These highly important substances are polyphenols, some of which exert even higher antioxidant action than vitamins [2,3].

The polyphenols represent a large group of at least 10,000 different compounds that contain one or more aromatic rings with one or more hydroxyl groups attached to them [4,5]. As secondary plant metabolites they are abundant in the majority of fruits and vegetables [5]. The most commonly occurring dietary polyphenols are flavonoids and phenolic acids [6]. In plants, polyphenols are generally involved in defense against different types of stress [7]. They offer protection against reactive oxygen and nitrogen species, UV light, pathogens, parasites and plant predators. Additionally, they contribute substantially to the organoleptic properties of plants, food and cosmetics [8]. Ancient civilizations have exploited their numerous biological effects for promotion and improvement of health for centuries [9]. In contrast, our knowledge of their properties has been until recently very limited [8]. Not so long ago polyphenols were even treated as non-essential anti-nutrients [10]. Nowadays ample evidence from copious studies exists of their antioxidative, anti-inflammatory and other various biological effects that exert in the prevention of various pathologies including cardiovascular diseases and cancer.

Prevention of disease by polyphenols is mainly due to their antioxidative properties, however, reversal of epigenetic changes can have strong effects as well [11]. It has been experimentally confirmed that polyphenols not only prevent various diseases but also impact on the disease propagation, suppress progression and even contribute to the healing process [5,12]. In addition, some polyphenols exert hormonal actions and inhibitory effects on bone resorption [3]. Therefore, polyphenols now represent the main target of cancer research as they show potential for becoming superior agents for preventing and treating various malignancies [2].

Advantages of polyphenols as anticancer agents are their high accessibility, low toxicity, specificity of the response and various biological effects. A combination of cytoprotective effects toward normal cells and cytotoxic effects toward cancerous cells thus represents the main advantage of polyphenols as anticarcinogenic agents [12]. Their role in carcinogenesis lies in the regulation of growth factor-receptor interactions and cell signaling cascades that can induce cell cycle arrest and impact on cell survival and apoptosis of cancerous cells [13]. Polyphenols mainly induce apoptosis through pro-oxidative action that is exerted instead of their anti-oxidative action depending on their concentration, target molecule/s and environmental conditions [2,14,15]. For that reason, they may interact differently depending on the cell type: healthy versus cancerous one. Additionally, polyphenols help to establish the body’s immune system by inhibiting angiogenesis necessary for tumor growth and act as anti-inflammatory agents [13]. In the final stages of cancer, polyphenols attenuate the adhesiveness and invasiveness of cells therefore reducing their metastatic potential [13]. However, the bioavailability of polyphenols represents a big hurdle as they only reach the target organs in very low concentrations. One auspicious solution for this problem represents nanoformulation of polyphenols that has brought some promising results [13]. On the other hand, it may lead to a problem of toxicity of specific agents when administered in high doses [2]. It was for example shown that some extracted polyphenols in high concentrations act even in the opposite way: instead of preventing cancer they can contribute to its formation and progression [4,16,17]. In contrast, some studies have shown that when in combination with other polyphenols an individual polyphenol can exert significantly enhanced chemoprotective and other favorable properties at considerably lower concentrations [18,19]. Synergistic action of polyphenolic mixtures additionally results in the concurrent impact on different disease pathways consequentially contributing to a faster and more effective healing [19]. Polyphenols can also suppress the side effects of certain therapies already used in the cancer treatment like chemotherapy and radiotherapy and enhance their action [18].

Dietary polyphenols are predominantly present in glycosylated forms with one or more sugar residue conjugated to a hydroxyl group or the aromatic ring (flavanols are one notable exception) [20,21]. This represents the main reason for their low absorption in the stomach as only aglycones and some glucosides can be absorbed in the small intestine, the rest are absorbed in the colon [20]. In comparison to the intestine, the colon does not readily absorb polyphenols. This also leads to longer absorption times, which can be up to 9 h [20]. The efficiency of colon-absorbed polyphenols reaches only 15%–20% of total polyphenol content being absorbed in the intestine [20]. Glucosides in food sources of polyphenols thus enable faster and more efficient absorption of polyphenols [20]. However, the aglycones of some isoflavones showed superior absorption to their glucosylated forms [20]. Isoflavones represent the best-absorbed polyphenols along with gallic acid, followed by catechins, flavanones, and quercetin glucosides [22]. On the other side, proanthocyanidins, galloylated tea catechins and anthocyanins are absorbed the least [22]. Conceptually, polyphenols are absorbed by passive diffusion [20].

Polyphenols are after intake subjected to three main types of conjugation: methylation, sulfation and glucuronidation [20]. The relevance of specific conjugation reactions is unclear and depends on the nature of the substrate and the ingested dose [20]. Some of these metabolic reactions contribute to their chemopreventive activities. Cancer protective effects of many polyphenols in different cancer models have indeed been shown regardless of their different mechanisms of action [4,23]. However, crucial factors that define the role of a specific polyphenol in target organs remain bioavailability and tissue levels [24].

The relative lipophilicity of polyphenols depends on the number of contained hydroxyl groups [3]. Interactions of polyphenols with lipids such as lipid cell membranes are limited to the polar region of the lipid bilayer [3]. Their penetration through the lipid membrane depends on their structure, where planarity is preferred [3]. Polyphenols are generally more hydrophilic than lipophilic owing to their phenolic nature [25]. Therefore, free polyphenols along with aglycones, glycosides and oligomers can be readily extracted by solvents such as methanol, ethanol, acetonitrile and acetone, or by their mixtures with water [25].

Since processes involving the use of organic solvents are known for their undesirable environmental and biological impact, intensive research is focused on new sustainable methods for the processing of substances [26]. Extraction by supercritical fluids (SCFs) is not harmful to food components and is environmentally safe, therefore applications of supercritical fluids represent a good alternative to other processing methods involving hazardous organic solvents and a high energy demand. SCFs may thus represent a substitute for conventional solvents and/or an aid to separation [27].

**Figure 1:**
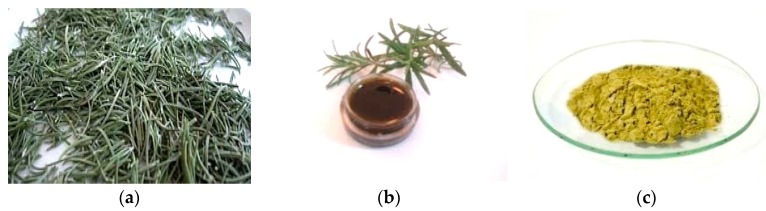
Rosemary (*Rosmarinus officinalis*) (**a**) leaves; (**b**) oil extract and (**c**) powder extract [29].

Health and safety benefits include the fact that the most important SCFs (SC CO_2_ and SC H_2_O) are non-cancerogenic, non-toxic, non-mutagenic, non-flammable and thermodynamically stable. Dense CO_2_ could be even used as a “green” processing medium, especially when organic solvents and high processing temperatures should be avoided [28]. Along with leaves and powdery extract, an oil extract of the culinary herb rosemary (*Rosmarinus officinalis*), obtained by supercritical extraction, is shown in Figure 1.

The purpose of this article is to review the bioavailability, antioxidative and anticarcinogenic properties of polyphenols, the methods for determining their antioxidative action and the currently used extraction methods for obtaining polyphenolic substances.

## 2. Extraction and Separation Methods for Phenolic Compounds

Numerous publications on the isolation and fractionation of phenolic compounds have appeared over the past two decades. The traditional methods for sample preparation, separation, detection, and identification are more and more frequently being replaced by advanced techniques [30].

In the first step, the proper extraction procedure has to be considered. The decision on the extraction method to be employed is influenced by the chemical nature of the substance, sample particle size, and also by the presence of interfering substances. Extraction time, temperature, solvent-to-feed ratio, the number of repeated extractions of the sample, as well as the choice of extraction solvents are the crucial parameters affecting the extraction yield. Solubility is influenced by both extraction time and temperature. A higher temperature simultaneously increases solubility and mass transfer velocities as well as decreases viscosity and surface tension of solvents contributing to a higher extraction rate [26,27]. For the elimination of the unwanted compounds such as waxes, fats, terpenes, and chlorophylls, additional steps may be introduced [31,32,33].

Phenolics can be extracted from fresh, frozen or dried plant samples. Before extraction, the material is pre-treated by milling, grinding, drying and homogenization. The selection of drying procedure impacts the total phenolic content. Freeze-drying retains higher phenolic content levels in plant samples than air-drying [34]. Phenolic extracts with a high anthocyanin content may also be obtained by using an acidified organic solvent such as methanol or ethanol. The goal is to select a solvent of a low viscosity in order to accelerate mass transfer [35].

### 2.1. Conventional Methods

Despite several disadvantages, liquid-liquid and solid-liquid extraction are still the most commonly used extraction procedures. For many years, the conventional techniques have been widely accepted, mainly because of their ease of use, efficiency, and wide-ranging applicability [36,37].

Such processes involve the use of conventional solvents like alcohols (methanol, ethanol), acetone, diethyl ether and ethyl acetate, often mixed with different proportions of water. There are several disadvantages of using these solvents: beside a possible hazardous effect on the human health, the residues of the solvents may also remain in the final products. This requires additional purification steps that are time-consuming and influence the total process cost. Additionally, by using pure organic solvents, very polar phenolic acids (benzoic, cinnamic acids) cannot be extracted completely. In such cases, mixtures of alcohol–water or acetone–water are suggested. Waxes, oils, sterols, chlorophyll are highly nonpolar compounds and may be extracted from the material by less polar solvents like dichloromethane, chloroform, hexane and benzene [36].

The yield and rate of polyphenolic extraction are related to the solvent characteristics. It has been observed that methanol is more efficient in the extraction of lower molecular weight polyphenols while aqueous acetone is a suitable solvent for the extraction of the higher molecular weight flavanols [38,39,40,41].

**Figure 2:**
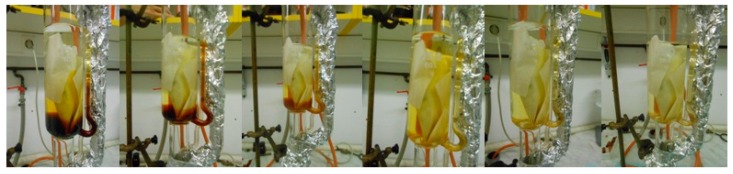
Soxhlet extraction from milled plant material [42].

However, many phenolic compounds are subject to degradation or undergo undesirable oxidation. The phenolic yield in the extracts is therefore significantly decreased. High processing temperatures should, therefore, be avoided. Conventional extraction is typically carried out at temperatures ranging from 20 °C to 50 °C. Temperatures exceeding 70 °C are undesired and lead to a rapid anthocyanin degradation. Long extraction times are yet another problem facing the conventional extraction procedures. In addition, the typical and most widely used conventional extraction methods, maceration and Soxhlet extraction, are known for their low efficiency and potential environmental hazards due to the high demand for organic solvents. In Figure 2 Soxhlet extraction from milled plant material can be seen.

Additional parameters controlling extraction kinetics are the sample matrix and particle size. Phenols may bind to other sample constituents such as carbohydrates and proteins [43]. In subsequent steps, these linkages may be hydrolyzed by the addition of enzymes, leading to the release of bound phenols [43].

Overall, the phenolic stability of the extract is influenced by acidic and alkaline hydrolysis and therefore by the pH of the sample as well as by the pH and polarity of eluents. Thus a pH of 4–5 was associated with increased stability of catechins and their isomers in comparison with alkaline and more acidic conditions [44].

### 2.2. Modern Extraction Techniques 

Due to problems associated with high processing temperatures and long processing times in conventional extraction procedures, there is an essential need to promote development and application of alternative extraction techniques for phenolic compounds. Possible alternatives represent ultrasound-assisted extraction, microwave-assisted extraction, ultrasound-microwave-assisted extraction, supercritical fluid extraction and subcritical water extraction that have recently gained a high interest [45] due to their simplicity, shorter extraction times and reduced organic solvent consumption.

Supercritical fluid technologies have been extensively investigated for selective isolation of antioxidants from natural material since the mild conditions avoid oxidation and/or degradation of labile compounds [46,47]. Recently, legal limitations for solvent residues and restrictions on the use of conventional organic solvents are becoming more and more rigorous, especially in the fields of food and pharmaceutical industry. Isolation/fractionation of special components using conventional production technologies (industry of oils and fats) is commonly replaced by alternative production technologies, carried out by processes with minimal environmental impact and low toxic wastes. Side-products are efficiently used during the process itself or in other industries and as the most important feature, products of higher quality and healthier nutrients are obtained. There is a large amount of papers dealing with the research on the supercritical extraction from different materials; determination and quantification of individual compounds, which shows the actuality of the topic, especially regarding the potential applications of natural compounds as additives. 

Since the high solubility of the compound of interest in the supercritical solvent is essential for the economy of extraction process, practical analyses shall verify if extraction using supercritical fluids is a suitable technique for the isolation of the target compounds [48]. Several parameters influencing solubility, mass transfer of target compounds in the supercritical fluid, and the resulting yield have to be considered [49]. Extract quality highly depends on the applied pressure and temperature which can significantly influence the composition of the final extracts. In addition, any pressure drop effect has to be evaluated and taken into account as well when optimizing parameters to obtain the best ratio between yield, solvent amount and extraction time. However, prior to initiation of extraction as the main step for the recovery and isolation of bioactive polyphenols from plant matter, the proper sample handling procedures have to be carried out. The first steps involve milling, grinding, and homogenization.

Considering the fact that supercritical fluid solvents represent intermediates between liquids and gases, by increasing the density of the fluid often an increased solubility is also achieved. The viscosity, which is similar to the viscosity of gases, enables better transport properties. The main advantage of supercritical fluids is the possibility to dramatically change the solvent characteristics near their critical point. Also, solvent selectivity represents an important solvent feature. It varies significantly with pressure and/or temperature. Often it is observed that a system with high solubility power possesses a low solvent selectivity. The later can be increased by adding a co-solvent [26].

Extraction of plant materials (like hop constituents, decaffeination of tea and coffee with supercritical CO_2_) constitute processes which represent some of the first applications of supercritical fluids and are already well established in the food sector. Natural substances for the application in cosmetic and pharmaceutical industry are also often obtained by using high-pressure tools. Supercritical fluid extraction technology has advanced tremendously since its inception and represents the method of choice in many food processing industries. Since it has been widely accepted as a clean and environmentally friendly “green” processing technique, it may be used as an alternative to the organic solvent-based extraction of phenolic compounds. Typically sterilized, contamination free phenolic compounds remaining in their chemically natural state may be obtained. Harmful components from nutraceutical products may be removed and enantiomeric resolution is possible. Removal of fat from foods, enrichment of vitamin E from natural sources, removal of alcohol from wine and beer, extraction and characterization of functional compounds as a consequence of the growing interest in the so-called functional foods represent some of the possible applications of supercritical CO_2_ extraction [27].

Overwhelmingly, supercritical CO_2_ represents the solvent of choice because of its easy penetration inside plant materials and its high solvent power. Recently, besides CO_2_, several alternative supercritical fluids have been proposed for extractive applications. For example, in the case of processing compounds of low polarity and low molecular weight, introduction of co-solvents and other supercritical fluids such as propane, argon and SF_6_ is carried out. Even water has been a topic of intense discussion, but its high critical temperature and pressure, related to the high energy consumption, together with the highly corrosive nature of H_2_O in the supercritical state, have limited its practical applications [8]. Subcritical water extraction has become an increasingly popular alternative technology in the extraction of phenolic compounds. In some cases, water is also added to the system as a co-solvent for the extraction of more polar compounds from aromatic plants. The dielectric constant of water is a strong function of both pressure and temperature. In the vicinity of the critical point, the dielectric constant and polarity may be easily fine-tuned by a small change of pressure. Due to the breakdown of intermolecular hydrogen bonds its polarity reduces under subcritical conditions. For instance, water has high polarity and a dielectric constant close to 80 at room temperature. By increasing the pressure at the temperature of 250 °C, the dielectric constant decreases significantly and becomes similar to the one of ethanol [50,51]. That means that the same solvent can be used to extract the inorganic and the organic components, respectively. 

The main advantage is that products produced in this fashion are solvent free, without the presence of co-products and that operational temperature is low [28]. The use of supercritical fluids for extraction of natural compounds at even higher pressures (over 70 MPa) than in conventional supercritical extraction will give new products from known plant materials such as isolation of less soluble substances. The main advantages of supercritical extraction over conventional methods are its simplicity, high extract quality, low extraction time and environmental friendliness due to water being used as the solvent.

### 2.3. Antioxidant Activity Assessment and Separation Methods

To evaluate the free radical scavenging capacity and the total antioxidant ability of single compounds and/or of complex mixtures, such as plants, foods and biological samples, several assays have been frequently used.

Widely established traditional spectrophotometric assays represent simple and fast screening methods for qualification of different classes of phenolic compounds in crude plant samples. Scavenging of the 2,2-diphenyl-1-picrylhydrazyl (DPPH) free radical has recently been applied to the phenolic compounds commonly present in natural tissues. This method represents the basis of a common antioxidant assay. The antiradical activities of various antioxidants are readily determined using the free radical DPPH, which shows a characteristic UV-Vis spectrum with an absorption band at 515 nm. The addition of an antioxidant is reflected in a decrease in absorbance proportional to the concentration and antioxidant activity of the added compound [52,53,54].

Because this method allows measurements of antioxidant efficiency at ambient temperature, the risk of thermal degradation of phenolic substances is eliminated. The reaction mechanism between the antioxidant and DPPH is, however, a strong function of the antioxidant conformation. This technique has been widely used to study antioxidant activity of several food commodities, such as oils, fruits, juices and wines [55,56,57,58,59].

The method has several advantages; it is easy to perform, known for a high reproducibility and comparable with additional methods such as 2,2′-azino-bis(3-ethylbenzothiazoline-6-sulphonic acid (ABTS), reduction of superoxide anion and inhibition of lipid peroxidation [60].

Classical methods, such as Folin-Ciocalteu and aluminum chloride complexation, are used to measure overall “total” phenolic and flavonoid contents after extraction [61]. However, the Folin-Ciocalteu reagent also interacts with other reducing non-phenolic substances, thus leading to an overestimation of the total phenolic content present. Reversed-phase high-performance liquid chromatography is commonly used to analyze different groups of phenols as well. In recent times, ultra-performance liquid chromatography has been applied to improve the analysis of phenolic compounds in various matrices [62]. Modern high-performance chromatographic techniques in combination with instrumental analysis present the “state of the art” for both, profiling and quantification of phenolic compounds [8].

High-performance liquid chromatography (HPLC) and gas chromatography (GC) are the two most frequently applied technologies to quantify phenolic compounds. Other relevant methods include the determination of the disappearance of free radicals using UV-Vis spectrometry. Currently, HPLC coupled with ultraviolet detection, electrochemical detection, mass spectrometry (MS) or particle beam/electron ionization mass spectrometry; gas chromatography coupled with MS, high-speed counter-current chromatography; chiral capillary electrophoresis or Fourier transform near infrared reflectance spectroscopy are the most commonly used methods for the determination of phenolic compounds. Hyphenated methods based on the HPLC separation, like HPLC-MS and HPLC-MS/MS, provide information about the molecular mass and structural features of compounds. They are considered to be more useful than other techniques in the separation, identification and quantification of phenolic content. Gas chromatography represents yet another highly effective technique for the separation, identification and quantification of several phenolic species, such as phenolic acids and flavonoids. The major drawback of GC analysis is that phenolic compounds are of low volatility, therefore, their derivatization is necessary [63].

Overall, gas chromatography and HPLC-MS are the preferred techniques for both separation and quantification of phenolic compounds. However, since these methods are both quite costly to purchase and maintain, many laboratories prefer to use the HPLC-UV detection, which is found to be less costly, comparably convenient to operate, and suitable for routine analysis [64].

## 3. Sources of Phenolic Compounds, Their Antioxidant and Anticarcinogenic Properties

### 3.1. Main Classes of Phenolic Compounds and Their Sources

**Figure 3:**
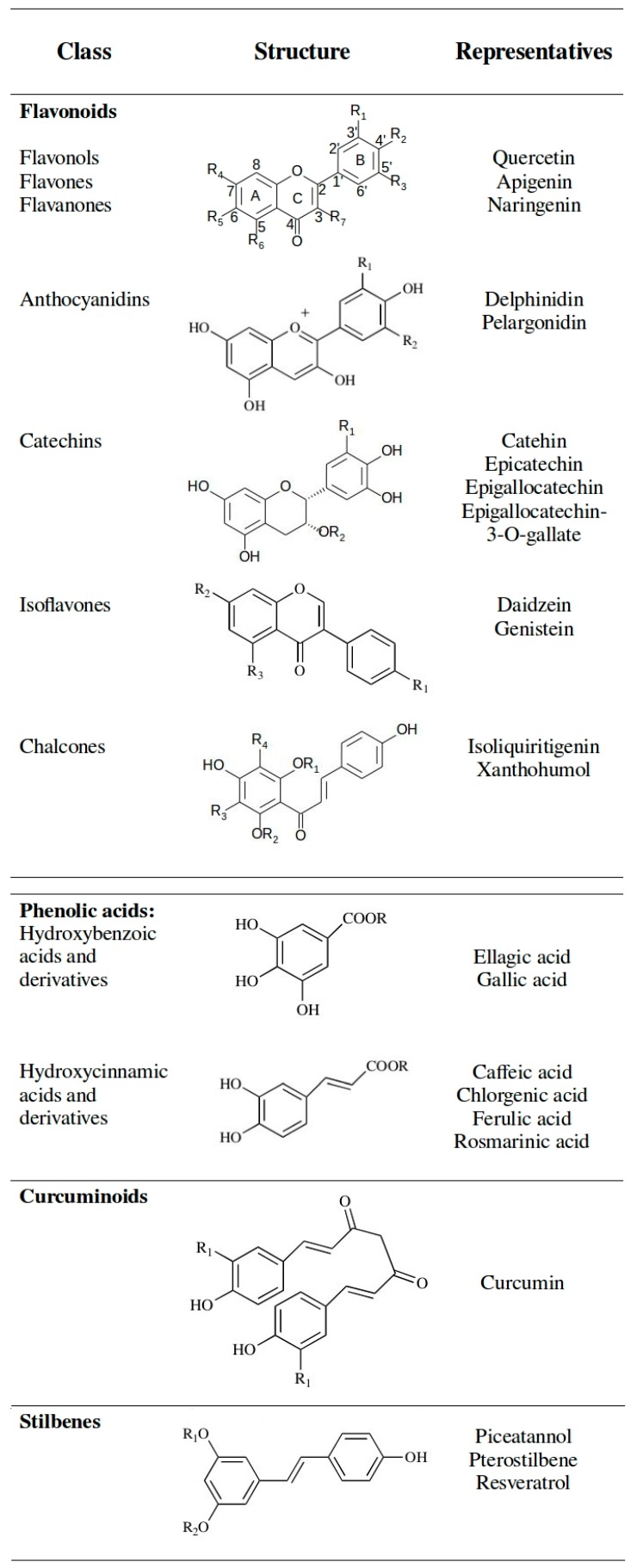

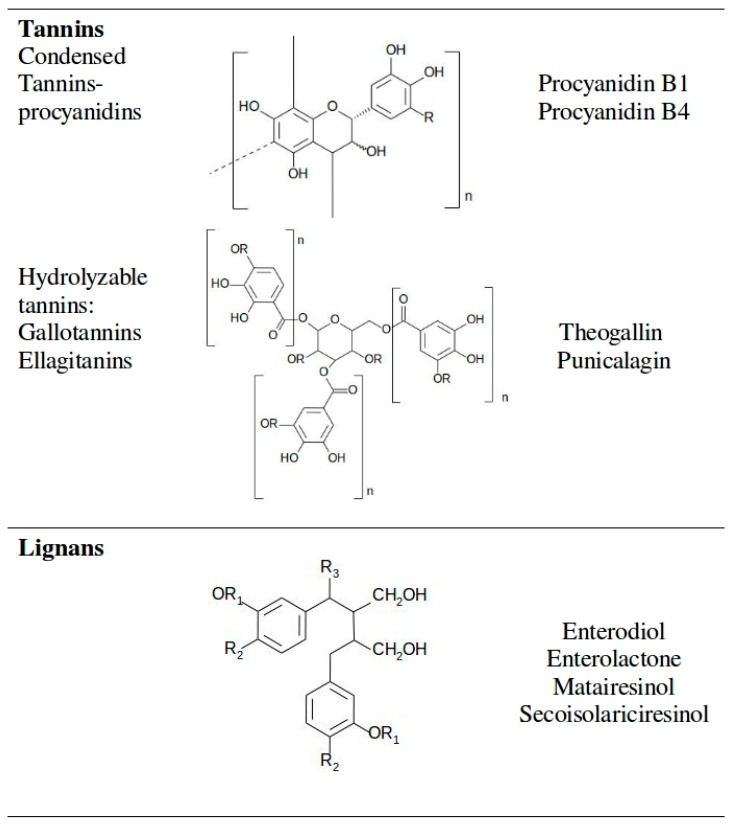
Polyphenolic classes with their basic chemical structure and typical representatives.

The variety of phenolic compounds reflects the very diverse biological functions carried out inside an organism. Polyphenolic compounds range from simple one benzene ring substances to molecules with several benzene rings, therefore a division into classes and subclasses is essential [6]. Among scientists, the most accepted is a separation into classes according to their chemical structure. Main classes of polyphenols are flavonoids, phenolic acids, stilbenes, lignans and tannins [21]. Less abundant in food, although of a high importance, is also the curcuminoids class [21]. Basic chemical structures and typical representatives of the most investigated polyphenolic classes are presented in Figure 3.

The main sources of polyphenols are fruits such as berries, grapes, citrus fruits, apricots, apples, plums, cherries, peaches and tropical fruits [4,65]. Other important sources are some popular beverages such as green and black tea, fruit juices, coffee, red wine, cocoa and beer along with various seeds, grains and nuts [2,21,66,67]. Among vegetables, polyphenols can be frequently found in onions, spinach, broccoli, cauliflower, artichoke, tomato, beans, soybeans, carrots, capers and olives [2,21,66,67,68,69]. Different spices and herbs such as clove bud, turmeric, celery, parsley, mint, rosemary, thyme, sage, dill, curry and ginger contain high levels of polyphenols as well [2,21,66,67,68].

Certain polyphenols, for instance, quercetin, are found in a plethora of plant products, while others can only be found in specific foods, like isoflavones in soy products [20]. Edible plants and plant products primarily contain complex mixtures of various polyphenols [20,70]. The content of polyphenols in plants varies significantly as it is a function of several parameters including genetic factors [67] (species differences [69]), environmental factors (climate, agronomic factors), manner of cultivation (organic or not), ripeness, storage (oxidation reactions) and culinary preparation [20]. The estimated daily intake of polyphenols is around 1 g which is significantly higher than the intake of all the other classes of dietary antioxidants and is for example approximately 100 times higher than the intake of vitamin E and carotenoids [2].

### 3.2. Flavonoids

Flavonoids represent the largest part of dietary polyphenols (up to 60%) [5]. Owing to their omnipresence and impressive biological functions/activities they continue to be thoroughly investigated as potential drugs or food supplements. Flavonoids consist of a diphenyl propane—flavone—skeleton with the three-carbon bridge between phenyl groups, commonly cyclized with oxygen [71,72]. The diversity among flavonoids calls for further division into subclasses. The most important subclasses include anthocyanins, chalcones, flavanols (catechins), flavanones, flavones, flavonols and isoflavones [5,6,11]. Their main representatives are collected in Figure 3. With the exception of catechins, flavonoids in plants are bound to sugars (glucose, galactose, rhamnose, xylose, rutinose, arabinopyranose, and arabinofuranose) in the form of ß-glycosides [71]. As already mentioned, the sugar residue determines their absorption [71]. Flavonoid glycosides are mainly located in the outer parts of the plant; whereas roots and tubers contain very low concentrations of flavonoids with some notable exceptions, like onions and licorice [20]. 

Some of the most common flavonoids are the flavonol quercetin, abundant in onion, broccoli, tea, and apples; the flavanol catechin found in tea and various fruits; the flavanone naringenin present in citrus fruits; cyanidin and anthocyanin giving color to many red fruits/berries (blackcurrant, raspberry, strawberry, blueberry, grapes etc.); daidzein and genistein—the main isoflavones in soybean [8,73].

A comprehensive literature review showed that there exists a variety of methods and strategies employed for the extraction of a particular class of flavonoids [74,75]. The main issue considering extraction of flavonoids (particularly glycosides) is that they can be easily degraded by enzymatic action when collected plant material is fresh or non-dried. It is therefore highly advisable to pre-treat the plant material in order to obtain dry, lyophilized or frozen samples. Another important feature is their polarity which significantly influences the selection of the extraction method. Isoflavones, flavanones, methylated flavones and flavonols as less polar flavonoids are extracted with organic compounds like chloroform, dichloromethane, diethyl ether or ethyl acetate. Flavonoid glycosides and more polar aglycones are extracted using alcohols or alcohol-water mixtures [76,77].

After pretreatment with hexane to remove lipids, ground plant material may be extracted in a Soxhlet apparatus using ethyl acetate or ethanol to obtain flavonoids. The main disadvantage of this method lies in high extraction temperature, therefore this approach is not suitable for heat-sensitive compounds. Sequential solvent extraction is used more and more often; the material is first extracted with dichloromethane. This step comprises isolation of flavonoid aglycones and other components of lower polarity. In the subsequent step, flavonoid glycosides and polar constituents are extracted using a suitable alcohol. Flavanone solubility is a strong function of the pH of water-containing solutions. Catechins, proanthocyanidins and condensed tannins as the most prominent representatives of flavan-3-ols, can often be extracted directly with water [78].

Flavonoids exert their antioxidative activity by effectively scavenging various free radicals (like superoxide anion and peroxynitrite), by regulating oxidative stress-mediated enzyme activity [12] and by chelation of transition metals involved in radical forming processes [79]. Other anticancerogenic properties include regulation of signaling pathways involved in carcinogenesis, interaction with proteins that control cell cycle progression and effective modulation of the wingless-related integration site (Wnt) signaling pathways in which most conventional therapeutics are ineffective [12]. Flavonoids can interfere with all three stages of cancer: the initiation, development and progression by modulating cellular proliferation, differentiation, apoptosis, angiogenesis as well as metastasis [70]. Moreover, chemopreventive effect of dietary flavonoids is quite specific as cancerous cells have shown to be more sensitive to polyphenol actions than normal cells [71]. Furthermore, flavonoids exhibit antibacterial, anti-inflammatory, anti-allergic and anti-thrombotic actions [79].

The inverse association of incidence of cancer, at all sites combined, with dietary intake of flavonoids, was observed in a study on Finnish men [24]. Another epidemiological study showed decreased cancer risk in the oral cavity, pharynx, larynx and esophagus [71]. However, there are also studies that did not find any relation between flavonoids intake and reduced cancer risk [71].

#### 3.2.1. Catechins

The catechins, a group of flavan-3-ols, has been comprehensively studied as their representatives form the major components of tea [80]. Catechins contain a benzopyran skeleton with a phenyl group bound to the 2-position and a hydroxyl group to the 3-position [80]. They exist in monomeric, oligomeric and polymeric forms and are not glycosylated [18]. Major sources of catechins are fruits, berries, cereal, nuts, chocolate, red wine and tea; estimated dietary intake is therefore very high (12–189.2 mg/day) [18]. In grapes and cocoa we mainly find (+)-catechin and (−)-epicatechin (EC), whereas in tea one mainly finds galloyl esters of catechins (gallocatechins) [24]. Representatives of both groups are strong antioxidants [10]. They can also act as pro-oxidants inducing H_2_O_2_ formation in order to provoke the apoptotic process in cancer cells [12]. However, the pro-oxidative action may induce reactive oxygen species (ROS) generation and have a deleterious effects on non-malignant cells [81]. Their anti-/pro-oxidative mode of action thus depends on the cell type, dose and time of treatment/exposure [12]. By inhibiting the activation of matrix metalloproteinase (MMP) enzymes MMP-2 and MMP-9, the ester-type catechins with a galloyl moiety inhibit the invasion of cancer cells [81]. Inhibition of lysyl oxidase (LOX) and cyclooxygenase (COX) activity by epigallocatechin-3-gallate (abbreviated as EGCG), epigallocatechin (EGC for short) and epicatechin-3-gallate (ECG), aside from modulating arachidonic acid metabolism, which importantly impacts cell growth, proliferation, tumor invasion and inflammation [81,82].

In addition to anticancer and antioxidative effects, catechins modulate lipid peroxidation [69,81] which affects weight gain and may inflict damage on the lipid layer of cells and consequently influence diseases such as diabetes, cardiovascular diseases and cancer [83]. Tea catechins also exert neuroprotective effects [82]. Catechins such as EGCG and ECG show the ability to modulate estrogen activity by influencing estrogen receptor (ER)-mediated gene expression through binding to ERα and ERβ [82].

Measured total peak plasma concentrations of EGCG, EGC, and EC (free and conjugated) were around 2 to 3 μM or lower [24]. Therefore, when extrapolating the results of the animal in vitro studies to humans caution is needed because in a majority of the in vitro studies significantly higher concentrations of polyphenols are used than those attainable in vivo.

The most prevalent tea polyphenol, EGCG, is presumed to exert the highest chemopreventive potential amongst antioxidative catechins [10,17,81,84]. EGCG presents up to about 10%–50% of the total catechin content [17,84]. It affects all three stages of cancer development, mainly by inhibiting a wide array of critical signal transduction pathways and by the activation of the redox-sensitive transcription factors [13]. Known inhibited signal transduction pathways include Janus kinase/Signal Transducer and Activator of Transcription (JAK/STAT), mitogen-activated protein kinases (MAPK), phosphatidylinositol-3-kinases/protein kinase B (PI3K/Akt), Wnt, and Notch [13]. EGCG also stimulates telomere fragmentation by inhibiting telomerase activity [13].

Various studies have demonstrated that EGCG inhibits carcinogenesis and also the growth of established cancers at various organ sites such as liver, stomach, skin, lung, mammary gland and colon [13]. Inhibited growth of cancerous cells can be attributed to inhibition of vascular endothelial growth factor (VEGF) production [1]. The antimutagenic, anti-inflammatory and anticarcinogenic effects of green tea can also be partially attributed to the strong antioxidative activity of EGCG that has been described in many in vivo and in vitro studies [81]. EGCG is able to arrest the cell cycle in phase G_0_/G_1_ and in the S-phase; both arrests can result in apoptosis [1,82]. Apoptosis can be induced by inhibition of nuclear factor (NF)-κB and activator protein 1 (AP-1) transcriptional activity as well as by p53 activation [10,65]. In vivo administration of EGCG reduces primary tumor growth and lung metastasis in mice bearing B16-F3m melanomas [82]. Direct binding to structural protein vimentin, can partially explain antiproliferative and antitumor promoting actions of EGCG [82]. An additional antiproliferative action of EGCG may be exhibited through suppression of human epidermal growth factor receptor 2 (HER2)/neu phosphorylation, inhibition of telomerase activity, inhibition of epidermal growth factor (EGF) mediated by its receptor EGFR activation and through the mediation of other multiple downstream signaling pathways [13,82]. On the contrary, EGCG may as well activate EGFR through pro-oxidant action, the mechanism of action thus depends on the circumstances [85]. Pro-oxidative action on multiple hydroxyl groups of EGCG and gallic acid in high doses, taken as dietary supplements, may, however, result in toxicity and carcinogenicity instead of chemoprevention [81].

Antiangiogenic effects, antimetastatic activity and suppression of invasion and proliferation of cancerous cells by EGCG may be a result of down-regulation of MMPs’ activity [10,13,81]. Additionally, EGCG is able to suppress cancerogenesis through anti-inflammatory action by downregulating interleukin 1 receptor type I (IL-1RI), by (NF)-κB inhibition [13] and by blocking the inhibition of Gap-junction intracellular communication (GJIC) [81]. As already mentioned, EGCG is also a strong inhibitor of lipid peroxidation [81]. 

It is presumed that the ability to modulate/reverse epigenetic changes is behind the majority of biological effects of polyphenols [11]. As cancer may be perceived as a manifestation of epigenetic changes and genetic predispositions, the reversal of epigenetic modifications by dietary polyphenols can prevent, suppress and even reverse carcinogenesis [86]. EGCG obviously possesses the ability to modulate/reverse all epigenetic changes: hypermethylation, histone modifications and changes in micro RNA (miRNA) expression [86]. It reverses hypermethylation of several known tumor suppressor genes such as p16, retinoic acid receptor (RAR), O6-methylguanine DNA methyltransferase (MGMT), and MutL homolog 1 (MLH1) in a concentration- and time-dependent manner; it is the most promising and potent modulator of histone markers in cancer cells; and it modifies the expressions of 61 miRNAs [86]. In human epidermoid carcinoma cells A431, EGCG (5–20 μM) treatment decreased global DNA methylation levels [71]. In parallel to epigenetic changes, EGCG’s inhibitory effect on histone acetyl transferase (HATs) enzymes may be beneficial in hormone-dependent prostate cancer as an androgen receptor (AR) and ERα are both regulated through acetylation by HATs [71]. EGCG has the ability to inhibit DNA and RNA synthesis and to alter DNA methylation through interaction with folic acid metabolism [86] and through inhibition of topoisomerase I activity [82]. In human liver cancer cells from HepG2 line, EGCG transcriptionally activates the phase II enzyme gene expression [1], which possibly results in the facilitated elimination of various carcinogens or their intermediates [24]. EGCG can even prevent UV radiation-induced photocarcinogenesis through regulation of several previously mentioned signaling pathways [81,87].

The reported beneficial effects of EGCG have led to its exploitation in clinical trials as a dietary supplement to improve endothelial function in humans with coronary artery disease to decrease the risk of cardiovascular diseases [88] and as a natural non-nucleoside inhibitor of DNA methyl-transferase 1 (DNMT1) [71]. Additionally, numerous clinical trials in connection with the anticancer protection effects of EGCG are ongoing as a part of green tea extract studies [65,89]. In order to improve the bioavailability of EGCG, nanoformulated particles of EGCG are being investigated. Nano-EGCG shows comparable anticancer, proapoptotic and antiangiogenic effects at a 10-fold lower dose than non-nano-EGCG [13]. Chitosan nanoparticles of EGCG provide a significant therapeutic benefit improvement against prostate cancer tumors compared to the free form [90]. Tumor inhibition was more pronounced and occurred even at a 6-fold lower dose compared to free EGCG [90]. Although structural modifications of (−)-EGCG have shown promising results toward enhanced anticancer effects, additional optimization and evaluation of EGCG analogs is needed in order to discover more potent, stable and specific novel anticancer agents [80].

#### 3.2.2. Flavonols, Flavones, Flavanones

Although flavonols, flavones and flavanones represent relatively diverse subclasses of flavonoids, they share some common biological activities. For example, several flavones such as apigenin, baicalein, luteolin and rutin, flavonols such as quercetin and kaempferol as well as flavanones such as hesperidin and naringin exert growth-inhibitory effects in different cancers: colon, prostate, liver, stomach, cervix, pancreas, breast, and leukemias [82]. 

The most extensively studied group among these subclasses are the flavonols, especially the main representative quercetin, which is not surprising considering that they are widely distributed in dietary plants [22]. The variability of flavonols is noteworthy, with about 380 flavonol glycosides and 200 different quercetin and kaempferol glycosides described to date [66]. However, their daily intake has been estimated to only 20–35 mg [22]. Glucosides of quercetin are more efficiently absorbed than quercetin itself, whereas the rhamnoglucoside (rutin) is less efficiently and less rapidly absorbed [22]. Therefore, onions that contain glucosides represent better sources of bioavailable quercetin than apples and tea, which contain rutin and other glycosides [22].

Flavanones represent a small group of compounds mainly found in citrus fruits and prunes [22,67]. The most important among the aglycone forms of flavanones (which are absorbed more rapidly) [22] are naringenin and hesperetin [91]. Nevertheless, significant amounts of flavanones in the aglycone form are rarely present in natural foods [22]. Glycosidic forms of flavanones are classified into two groups: neohesperidosides and rutinosides. The bitter taste of bergamot, grapefruit and bitter orange juices mainly comes from neohesperidosides such as naringin, neohesperidin and neoeriocitrin [91]. Rutinoside flavanones hesperidin, narirutin and didymin are on the other hand present in bergamot, orange, mandarin and lemon juices [91].

A majority of flavonols, flavones and flavanones possess antioxidative activity [91]. However, the absence of the hydroxyl group at position 3 in flavanones and flavones decreases their antioxidant ability, similar to the absence of the catechol structure in the B-ring [91]. On the other hand, the 2,3-double bond makes the structure more reactive—for this reason apigenin is a moderate antioxidant compound—while naringenin has no reported activity against superoxide ion [91]. Flavonoids exert antioxidative activity in a hydrophilic environment. In a lipophilic environment, molecules like neohesperidin, hesperetin, didymin and isosakuranetin display a reduced antioxidative capacity, while naringin, narirutin, naringenin, neoeriocitrin and heridictyol even show reversed behavior, becoming prooxidants [91]. Additionally, luteolin, kaempferol, quercetin, and naringenin possess the ability to inhibit the estrogenic action of 17-β-estradiol through competitive binding to the estrtogen receptor (ER) [92]; baicalein inhibits androgen receptor expression [82]. Quercetin, baicalein and apigenin arrest cancerous cells at the G_2_/M phase of the cell cycle, baicalein also induces arrest in the G_1_/S phase and apigenin causes the G_0_/G_1_ arrest as well [13,93]. All arrests of the cell cycle consequentially induce apoptosis [83]. Moreover, baicalein and kaempferol trigger apoptosis through inhibition of B-cell lymphoma 2 (Bcl-2) expression [82]. Certain flavonoids also impact DNA replication through inhibition of reverse transcriptase. Prerequisite for this action is the presence of both the unsaturated double bond between positions 2 and 3 of the flavonoid C ring, and of the three hydroxyl groups introduced at positions 5, 6, and 7 (i.e., baicalein) [82].

A number of extraction methods have been developed in recent years such as microwave, ultrasound-assisted extractions, and techniques based on the use of compressed fluids as extracting agents, such as subcritical water extraction, supercritical fluid extraction, pressurized fluid extraction or accelerated solvent extraction. The conventional maceration technique, microwave and ultrasonic extraction in combination with alcohol solvent were the most efficient extraction techniques [8]. In the following sections, the anticancer properties of apigenin, quercetin, fisetin, naringenin, naringin and hesperetin are presented in more detail as they show potential in cancer prevention and treatment and are relatively abundant in our diet.

##### Apigenin

Apigenin is a naturally occurring plant flavone abundant in common fruits and vegetables such as grapefruits, plant-derived beverages, parsley, onions, chamomile, oranges, tea and wheat sprouts [93]. The most frequent source of consumed apigenin is chamomile in the form of herbal tea [93]. Owing to its potent antioxidant, anti-mutagenic, anti-inflammatory, anti-viral and purgative effects, a plethora of studies has focused on possible chemopreventive effects of apigenin [93]. In addition, it has low intrinsic toxicity and shows striking effects on normal versus cancer cells compared to other structurally related flavonoids [13]. Both in vitro and in vivo studies suggest that the inhibition of histone deacetylases by apigenin is responsible for mediation of cancer growth inhibitory responses [93]. As already mentioned, apigenin is also able to induce apoptosis, moreover, it can induce it through several different pathways [13,93]. Anti-angiogenic action of apigenin is similar to that of EGCG, as well as inhibition of tumor growth, suppression of progression and invasion of tumor and prevention of metastasis through altering Bcl-2 associated X protein (Bax)/Bcl-2 ratio, MAPK and PI3/Akt signaling and through modulation of insulin-like growth factor (IGF), VEGF, MMP’s and transforming growth factor TGF-β1 [13,93]. Apigenin and other hydroxyflavones are shown to potently inhibit the expression of cytokine-induced adhesion molecules by endothelial cells. Apigenin also reduces proliferation by lowering cyclin D1 (regulator of the cell cycle) [94]. Apigenin exerts its anti-inflammatory effects by blocking interleukin 1α-induced prostaglandin production, as well as the production of the cytokines interleukin-6 and interleukin-8 [94]. Finally, apigenin is known for its ability to reduce oxidative DNA damage [93].

##### Quercetin

Quercetin represents the main antioxidative flavonol in the human diet as it is contained in various fruits, vegetables, beverages, nuts, seeds, flowers and bark, being particularly abundantly in onions (0.3 mg/g fresh weight) and tea (10–25 mg/L) [24,70,81]. Therefore, quercetin is one of the most studied anticancer phenolic compounds known to date [13]. Amongst the more than 170 different quercetin glycosides that have been identified, quercetin usually occurs as *O*-glycosides with d-glucose as the most frequent sugar residue [24]. In humans, quercetin glucosides show much higher bioavailability than quercetin rutinosides, suggesting that the glucosides are actively absorbed in the small intestine [24]. The uptake of quercetin can vary between 5 mg/day and 40 mg/day however it may as well increase up to 10-fold, if the diet includes fruits and vegetables particularly rich in this compound, such as onions, apples and strawberries [91]. 

At chemopreventive or pharmacological doses, quercetin has been shown to modulate almost all of the different hallmarks of cancer [91]. Moreover, quercetin exhibits a dose-dependent inhibitory effect on cell growth in various types of cancer according to numerous in vitro and in vivo studies [13]. Inhibition of several chemically induced tumors by quercetin has been also reported in different studies [10,24,91]. Even when administered in the prenatal period it decreases the susceptibility to lung cancer [91]. Anticancer effects of quercetin on tumor cells are exerted through inhibition of cell division by interference with the cell cycle components, like cyclin D1 and by induction of apoptosis, necrosis and even autophagy [10,13,71,95]. Quercetin can trigger apoptosis in vivo through several mechanisms such as G_1_, S and G_2_/M phase arrests, a p53-dependent mechanism and through the modulation of MAPK and PI3K/Akt pathways [13,82,95]. Additionally, quercetin inhibits proliferation; it has also reduced tumor incidence in mice by 76% and tumor multiplicity by 48% [10,95]. Being a functionally pleiotropic molecule, quercetin has an impact on multiple intracellular targets and affects different cell signaling processes usually altered in cancer cells, with limited toxicity to normal cells [91]. Simultaneously targeting various pathways may help to kill cancer cells and slow drug resistance onset [91]. Like EGCG, several of the listed effects could be a consequence of the influence of epigenetic modifications [11,71,86]. 

Quercetin acts as a powerful antioxidant since it possesses all the structural functionalities that enable the maximum radical scavenging potential [8,91]. Even by the addition of micromolar concentrations quercetin can lower H_2_O_2_-induced ROS in cell lines [91]. The 3-glycosylation reduces the antioxidative activity when compared to corresponding aglycones [8]. Although quercetin is a small molecule, it is easily oxidized and thus possesses pro-oxidant activity that could result in enhancement of tumors, fortunately, such effects have not yet been observed in human studies [8,24]. Quercetin inhibits DNA damage induced by cooking oil fumes, presumably by increasing stabilization of DNA secondary structure [81,82]. On the other hand, prolonged treatment of DNA with quercetin solutions led to an extensive disruption of the double helix through pro-oxidative action (formation of H_2_O_2_) [10,82]. The hydrophobic core of quercetin may be the cause of its interaction with DNA, however, it binds to DNA mainly through electrostatic interactions [82]. Quercetin additionally represses COX-2 mRNA and protein levels that also contribute to genomic instability [81]. Compared to catechin and epicatechin, quercetin is a significantly more potent suppressor of COX-2 transcription as it can affect it by inhibiting the p300 signaling and by blocking the binding of multiple trans-activators such as activating transcription factor 4 (ATF4, former CREB2), c-Jun, CCAAT/enhancer-binding protein beta (C/EBPβ) and NF-κB to COX-2 promoter [71,82]. Moreover, quercetin features inhibition of nitric oxide (NO) and inducible nitric oxide synthases (iNOS) protein expression without affecting iNOS mRNA expression [82].

Repression of carcinogenesis by quercetin is also due to blocking of the tetradecanoyl phorbol acetate (TPA)-induced inhibition of GJIC, inhibition of telomerase and inhibition of DNA polymerase-β [8,81,82,96]. Functional GJIC is essential for maintaining homeostasis in multicellular organisms [8,81]. Similarly to EGCG, quercetin blocks proliferation, invasion, migration, metastasis and angiogenesis mainly through inhibition of several MMPs [5,81,82]. Although quercetin is able to markedly decrease the multiplicity of papillomas and the incidence of carcinomas after topical application to mouse skin, when orally administered it did not prevent UVB-induced skin carcinogenesis, probably due to the bioavailability problem [24]. Besides the described effects on carcinogenesis, quercetin is also an apt inhibitor of lipid peroxidation activity and exhibits anti-infective and anti-replicative activity against certain viruses [81,97]. However, regular diet cannot provide adequate amounts of quercetin compatible with several listed chemopreventive effects, although it is relatively easy to increase its total concentrations in plasma by the intake of quercetin-enriched foods or supplements [91]. 

##### Fisetin

Along with quercetin, myricetin and kaempferol, fisetin belongs to the flavonol subgroup of flavonoids [93]. Fisetin can primarily be found in fruits and vegetables, such as strawberries, apples, persimmons, grapes, onions and cucumbers [93]. In vitro and in vivo studies have shown that fisetin is able to induce apoptosis, cell cycle arrest, to inhibit androgen signaling and tumor growth [93]. Additionally, fisetin shows antiproliferative effects on human prostate cancer cells and selectively decreases the viability of cancerous cells with minimal effects on normal epithelial cells [93]. Therefore, fisetin has a high potential for becoming an effective agent against prostate cancer and possibly other cancer types as well [93].

##### Naringenin, Naringin, Hesperetin

A majority of flavanones is found solely in various citrus fruits. Even though larger quantities of polyphenols are found in the seeds and peels of citrus fruits, considerable amounts are also present in the fruit itself and in juices [91]. Lemon is the most important source of hesperidin, whereas glycosylated naringin is mainly found in bergamot, lemon, mandarin seeds and lemon peels, however, it is not present in the juices of these fruits [91]. Among several flavonoids contained in grapefruit juice, naringenin qualitatively and quantitatively represents the principal component [98]. 

Orange juice or naringin-supplemented diet together with grapefruit juice significantly delay tumor development and reduce the incidence of mammary fat pad tumors in mice by more than 50% [91,94].

Moreover, naringin inhibited the in vivo development of 7,12-dimethylbenz[*a*]anthracene-induced mammary tumors in Sprague-Dawley rats [69]. It also plays an important role in regulating antioxidative capacity by increasing superoxide dismutase and catalase activities and by up-regulating the gene expressions of superoxide dismutase, catalase and glutathione peroxidase in cholesterol-rich diet-fed rabbits [91]. Naringin blocks H_2_O_2_-induced cytotoxicity and apoptosis and thus protect normal cells from oxidative stress, however in tumorigenic cells apoptosis is the key mechanism of cancer suppression [91]. 

Naringenin significantly reduces lung metastases in mice and increases their survival by improving the immunosuppressive environment through down-regulating TGF-β and reducing regulatory T cells [7]. Naringin also inhibits mammary carcinogenesis, but the rats in the study group showed lower weight gains compared to the control group, and this may have had an influence on carcinogenesis [94]. Additionally, naringenin stimulates DNA repair following oxidative damage in human prostate cancer cells and inhibits proliferation of certain colon cancer cell lines [91]. The citrus flavonoids hesperetin and hesperedin were among the most potent inhibitors of malignancy, inhibiting malignant transformation almost completely (98%) at very low concentrations (1 mM) [69]. Quercetin and rutin at the same dose inhibit carcinogenesis by 50% and 30%, respectively [69]. Hesperetin, in comparison to quercetin, shows only anti replicative activity against some viruses [97]. 

Several citrus flavanone glycosides, the most active being hesperidin and naringin, and their aglycones, administered at a dose of 45 mg/kg daily, exert anti-inflammatory effects in the rat granuloma pouch model [94]. Lymph node metastases and lung metastases were the least present in the orange and grapefruit juice fed groups of mice, followed by the groups given naringin, hesperidin or naringenin [94].

#### 3.2.3. Isoflavones

Isoflavones are naturally occurring phytochemicals of the flavonoid class and have been called ‘phytoestrogens’ due to their estrogen-like effects [88]. The predominant source of isoflavones are legumes, more specifically soy products [70]. In Asia, soy foods are abundant in the diet, whether fermented, like soy paste, or unfermented, like tofu and soy flour. Consequently, the daily consumption of isoflavones there is relatively high. On the contrary, in the western world, the source of soy isoflavones and proteins are mainly baked products, where they are present as fillers of extenders, and various dietary supplements [88]. For binding to the estrogen receptor, a distinct feature of phytoestrogens, with few exceptions, represents the phenolic ring [88]. Phytoestrogens with the phenolic ring can act as estrogen agonists or at higher concentrations even as estrogen antagonists [10,70,88]. Main isoflavones in soybeans predominantly present in the form of conjugated glycosides are genistein and daidzein (approximately 1 mg/g of dry soybeans) [24,88]. In humans, plasma or serum levels of genistein originating from soy food ingestion range from less than 1 µM up to about 5 µM [88].

Among all polyphenols, isoflavones are the best absorbed in humans. Their bioavailability predominantly depends on gut microflora activity [22,70]. Therefore, isoflavone absorption and pertaining beneficial effects may vary considerably between individuals [70]. It has for example been demonstrated that the injected genistein is more effective in inhibiting transplanted tumors than if consumed orally [70]. Although systemic bioavailability is significantly higher for genistein than for daidzein [22]. Bioavailability and absorption of isoflavone aglycones and glucosides are still unclear as studies show contradictory results. Therefore, it is still unknown which of the two is absorbed better. Some studies even found no significant differences in the absorption efficiency between aglycones and glycosides [22]. However, equol production is significantly higher after ingestion of daidzin (glucoside) than after ingestion of daidzein (aglycone) [22]. Bacterial isoflavandiol metabolite of daidzein equol is shown to be more estrogenic than its precursor daidzein in many in vitro studies and in animal models [22,99]. Intervention studies also suggest that the maximal clinical response to soy proteins is equol-dependent [88]. 

Animal studies provide evidence for both beneficial and harmful biological effects of soy/isoflavones. Sadly, dose, the timing of administration and type of isoflavones required to produce either benefit or harm are relatively unknown [88]. Beneficial effects of soy and associated isoflavones include antioxidative, anti-tumor and anti-lipogenic activities [88]. Genistein is an effective scavenger of superoxide and peroxynitrite radicals generated from enzymatic and nonenzymatic systems [81]. Moreover, several studies have revealed that genistein exhibits protective effects against DNA damage caused by ROS and reactive nitrogen species either alone or in combination [81]. Meta-analysis of 38 clinical studies revealed that consuming 47 g of soybean protein daily, with active components genistein and daidzein, decreases total cholesterol, low-density lipoprotein (LDL) cholesterol and triglycerides by around 10% with a greater response in subjects having a higher baseline cholesterol [70]. Some studies revealed that in women with normal cholesterol this activity is not observed, therefore the action depends on conditions [70]. 

Soy phytoestrogens exert several beneficial effects toward osteoporosis, cardiovascular diseases such as arteriosclerosis, and modest blood pressure–lowering effects, mainly due to isoflavones [17,70]. Multiple lines of compelling evidence from a number of epidemiological studies report an inverse correlation between dietary soy consumption and the risk of certain cancer types [3,100]. Although soybeans contain a number of ingredients with demonstrated anticancer activities, genistein has proven to be the most important agent and was therefore extensively investigated and studied [17,100]. Various studies have revealed that genistein indeed does exhibit potent anti-invasive and anti-metastatic activities toward the breast, prostate, lung, colon, melanoma, ovary, sarcoma, liver, gastric, oral, pancreas and brain cancer cell lines [8,10,24,100]. Moreover, genistein demonstrates inhibitory effects against mammary tumorigenesis by soybeans or soybean products mainly attributable to genistein as daidzein is less effective in reducing tumor multiplicity [70]. Inhibition of mammary, prostate and breast cancer by genistein is presumably due to its modulation of estrogenic activity [24,70]. Genistein inhibits growth and enhances apoptosis of LNCaP (human prostate adenocarcinoma) cells, partially due to down-regulation of the expression of EGF and HER2/Neu receptors, inhibition of Akt activity and due to Akt-mediated NF-κB activation [1,70]. Additionally, genistein reduces the volume of transplanted murine bladder cancer, reduces angiogenesis and increases apoptosis presumably through the G_2_/M cell cycle arrest [70]. On the other hand, genistein is reported to increase proliferation of breast epithelium as well as the noninvasive and total adenocarcinoma multiplicity, but to have no effects on the multiplicity of invasive adenocarcinoma [24,70]. A diet containing 1 g/kg of genistein increases the progression of mammary adenomas [10]. Accordingly, applications of isoflavones as chemopreventive agents in humans, because of the possible estrogenic activity of high doses represent a concern, especially in premenopausal women and infants [24].

Even though several in vitro studies have shown that genistein apparently induces DNA demethylation through DNMT inhibition, animal studies have shown rather the contrary increased DNA methylation following the treatment [71,86]. A diet with soy isoflavones (genistein and daidzein) feed to mice results in an advancement of sexual maturation in female pups as well as in suppression of normal gender differences in the DNA methylation patterns of tissue-specific methylated genes mostly through induction of hypermethylation in some genes of female mice [86]. In addition to modification of methylation, genistein possesses other epigenetic activities like histone modifying activity (highest among isoflavones) and regulation of the miRNA expression as well [11,71,86]. The latter is potentially important in designing novel therapies for pancreatic cancer as it leads to the reversal of epithelial-mesenchymal transition phenotype thus preventing metastasis [86]. Some clinical trials have also suggested that soy isoflavones may benefit some patients with prostate cancer [3]. Other clinical trials on the investigation of the efficacy of soy products and genistein in cancer prevention are still ongoing [3].

A recent study shows that genistein prevents the TPA-, hydrogen peroxide- and phenazine methosulfate-induced inhibition of GJIC [81]. Additionally, genistein effectively suppresses the COX-2 promoter activity [71,81]. Potent inhibition of the production of certain cytokines and eicosanoid biosynthesis suggests that genistein modulates inflammatory responses that are commonly involved in the promotional stage of cancer [3,17]. Moreover, genistein inhibits the activities of tyrosine protein kinase (through influence on proliferation), topoisomerase II and ribosomal S6 kinase in cell cultures by stabilizing a cleavable topoisomerase-DNA complex and by modulating mRNA translation in vitro, which may lead to protein-linked DNA strand breaks, cell growth suppression, differentiation and induction of several malignant cell lines [10,17,88]. 

#### 3.2.4. Anthocyanidins

Anthocyanidins naturally occur as glycosides named anthocyanins, thus anthocyanidins’ biological function is foremostly attributed to the latter [7]. As universal plant colorants, anthocyanidins represent the most prominent members of the bioflavonoid group of phytochemicals [9,101]. Over 600 anthocyanidin molecular structures have been identified to date, foremostly because they provide the red, purple and blue hues of various fruits, vegetables, cereal grains and flowers [9]. The color of anthocyanidins is pH dependent [8]. Due to their multiple biological effects, anthocyanins may play a role in enhancing the health-promoting qualities of foods [9]. Therefore, they are of immense interest to the food (colorant) industry [9]. The main sources of anthocyanidins include teas, honey, wines, fruits such as apples, berries; vegetables like beets and onions; nuts, olive oil, cocoa and cereal [8,101]. Anthocyanins are primarily found in the flowers and fruits of various plants and only to a lesser extend in leaves [8,9]. Major representatives of the anthocyanidin group constitute delphinidin, pelargonidin, malvidin, cyanidin and petunidin [9,101]. Estimated daily intake of anthocyanidins varies between 500 mg to 1 g, however, it can easily reach several g when an individual is consuming flavonoid supplements such as grape seed extracts, ginkgo biloba, or pycnogenol [101]. Anthocyanins are very poorly absorbed, though, all of the metabolites might not have been identified resulting in an underestimation of their bioavailability [22]. Bioavailability of anthocyanidins and anthocyanins still remains a challenge for further investigation that would explain controversy in the available results [101]. The main reason for this is presumably the inability of scientists and medicinal practitioners to track the metabolic progress of anthocyanins after ingestion, due to the plethora of metabolic breakdown products that are rapidly produced in situ [101].

Anthocyanidins have been long used as medicinal agents in various folk medicines throughout the world, however, measurable pharmacological properties of isolated anthocyanin pigments have been conclusively verified only in the recent years [9,101]. Although novel scientific research clearly suggests that anthocyanidins use several different mechanisms of action to reach health-beneficial biological effects, the most highly publicized remain free-radical scavenging and antioxidant capacity [8,9,101]. Anthocyanidin isolates and anthocyanidin-rich mixtures of bioflavonoids have demonstrated protection against DNA cleavage, estrogenic activity (altering the development of hormone-dependent disease symptoms), enzyme inhibition, boosting production of cytokines (thus regulating immune responses), anti-inflammatory activity, lipid peroxidation, decreasing capillary permeability and fragility, and membrane strengthening [8,9,101]. Consequently, they are able to reduce the risk of cardiovascular diseases and with the anticancer and chemoprotective properties induce prevention and treatment of several tumors [8,100]. In vitro and in vivo research trials have demonstrated anthocyanidins’ marked ability to reduce cancer cell proliferation and to inhibit tumor formation [8,9,101]. It is suggested that the main mechanisms for the prevention of carcinogenesis include inhibition of cyclooxygenase enzymes, potent antioxidant potential and blocking the activation of an MAPK pathway [9,100,101]. 

The degree of anthocyanins’ bioactive properties strongly depends on their chemical structure (position, number, and types of substituents) and on their intracellular localization [101]. Anthocyanidins possess numerous additional biological effects such as enhancing (night) vision, inhibition of body weight and adipose tissue increases; therefore they can aid in the prevention of ophthalmological diseases, obesity and diabetes [101]. Moreover, anthocyanidins have been credited with the capacity to modulate cognitive and motor functions, to enhance memory; consequently, they play an important role in the prevention of neurological diseases, possibly owing to their high bioavailability in endothelial cells [101]. The general antimicrobial activity of anthocyanins has been well established, including significant inhibition of aflatoxin biosynthesis [101]. 

Anthocyanins can be present in plant tissues as different chemical species making them a unique example among plant phenolics. Solvents containing mineral or organic acids are widely applied for the extraction of anthocyanins from plant organs. Indeed, in the range of low pH, anthocyanins predominantly appear in the form of flavylium cation that contributes to their typical reddish color in aqueous solutions. By altering the pH, the flavylium cation is converted into other species. In a highly acidic medium, the flavylium cation form is red and stable. Facile hydrolysis of anthocyanin acetates occurs upon the exposure to trace quantities of mineral acid during the extraction processes, and some authors working on chemotaxonomic studies have reported that the extraction of some acylated anthocyanins under acid conditions may cause their partial or total hydrolysis. Moreover, the use of acidic solvents for the extraction of anthocyanins may lead to the generation of anthocyanidins from flavanols and proanthocyanidins. These techniques have been found to have a relatively high environmental impact, besides they are very time-consuming. Supercritical fluid extraction has been recently successfully applied for the extraction of valuable compounds from grape (*Vitis labrusca* B.) peel by modifying process parameters such as temperature, pressure and modifier concentration. Influence of extraction temperature and pressure have been found significant on all responses [76,102].

#### 3.2.5. Chalcones

Another important class of naturally occurring flavonoids represents chalcones which are also metabolic precursors of certain flavonoids and isoflavonoids [103]. Chalcones are particularly abundant in hops and therefore in beer, also in fruits like citruses and apples; in certain vegetables such as shallot, tomatoes, potatoes and bean sprouts and in various plants and spices (licorice, cardamom) [103,104]. 

Plants containing chalcones have been employed in traditional herbal medicine for centuries [103,104]. For that reason, chalcones caught the attention of the scientific world that was later justified as this class of flavonoids exerts a wide spectrum of biological activities. Chalcones possess antioxidative, antibacterial, anti-inflammatory, anticancer, cytotoxic, and immunosuppressive potentials [104]. Most of their anticancer activity might be attributed to molecular alterations such as induction of apoptosis, DNA and mitochondrial damage, inhibition of angiogenesis, tubulin inhibition, kinases inhibition, and also drug efflux protein activities [103]. Chalcones trigger apoptosis through different cell death pathways, not just by blocking the process of cell division [103]. Compounds belonging to this class of flavonoids have been also shown to interfere with each step of carcinogenesis, including initiation, promotion and progression [104]. Observed bioactivities of chalcones that provide various anticancer activities essentially depend on their structure with the most important features being heterocyclic rings, hydroxyl and other substituents on both aryl rings [103,104]. It has been shown that predominantly chalcones with hydroxyl and prenyl substituents exhibit important antioxidant properties, specifically the induction of quinone reductase activity that is responsible for detoxification [104]. Hydroxyl derivatives of chalcone also possess more potent anti-proliferative properties than other chalcone derivatives [104]. On the other hand, glycosidic substituents on the aromatic rings cause impaired abilities for suppressing proliferation toward cancer cells compared with corresponding aglycones [104]. Unlike in the other flavonoid classes, numerous compounds among (dietary) chalcones appear to show activity against cancer cells [104]. 

In comparison with other polyphenols, chalcones have some strong advantages as anticancer agents such as poor interaction with DNA, low risk of mutagenicity, xanthohumol is even devoid of any estrogenic activity [103]. Together with higher selectivity toward leukemic cells compared to nontumoral cells and various biological activities even in nanomolar concentrations, chalcones pose an enormous potential for becoming novel anticancer pharmaceuticals [103].

Xanthohumol is the main prenylated chalcone, predominately found in hops and consequentially in beer [100]. In enzymatic assays, xanthohumol was able to modulate the activity of several enzymes, scavenge reactive oxygen and nitrogen oxide species production and decrease inflammation by inhibiting COX-1 and COX-2 activity [6,104]. Although xanthohumol does not possess estrogenic activity (it even demonstrates potent antiestrogenic properties), it exerts apoptotic and anti-invasive effects on human breast cancer cell lines together with suppression of tumor growth [6,100]. It seems to be the most potent antiproliferative agent against prostate cancer cells among chalcones [104]. Xanthohumol belongs to a class of so-called multi-functional compounds that target more than a single cellular process, thus affecting various cancer stages [104]. Despite the fact that their mechanisms seem to be nonspecific, they might nevertheless selectively target one distinct regulatory protein that modulates many downstream signaling pathways and thus initiate a cascade of cellular events that lead to the described spectrum of effects [104]. 

Similarly to xanthohumol, isoliquiritigenin, a chalcone found in licorice, shallot and bean sprouts, is a potent antioxidant with anti-inflammatory and anti-carcinogenic activities like inhibition of metastasis and invasiveness [100]. Isoliquiritigenin even inhibits basal and EGF-induced cell migration, invasion and adhesion in a dose-dependent manner [100]. 

### 3.3. Phenolic Acids

Representing one-third of consumed phenolic compounds, phenolic acids are a highly important class of polyphenols [24]. As it can be seen in Figure 3, phenolic acids are divided into two major groups: hydroxybenzoic acids and hydroxycinnamic acids with their respective derivatives [24]. In foods, these compounds are present as esters, either soluble and accumulated in vacuoles or insoluble as cell-wall components [24]. Phenolic acids are present in leguminous plants: some vegetables like spinach, broccoli and kale; in berry fruits, apples; some beverages like coffee, tea, citrus juices, wine, beer; in cereal brans and in olive oil [22,66,73,82]. One of the most common phenolic acids is caffeic acid, present in many fruits and vegetables, most often esterified with quinic acid as in chlorogenic acid, which is the major phenolic compound in coffee [73]. Another common phenolic acid is ferulic acid, which is present in cereal and is esterified to hemicelluloses of the cell wall [73].

Phenolic acids have antioxidant activity as chelators and are free radical scavengers with special impact on hydroxyl and peroxyl radicals, superoxide anions, and peroxynitrites [93]. Caffeic, sinapic, syringic, protocatechuic, ferulic and 3,4-dihydroxyphenylacetic acid; found specifically in virgin olive oil, decrease the proliferation of breast and prostate cancer cells in a time- and dose-specific manner [82]. Caffeic acid, ellagic acid, chlorogenic acid, and ferulic acid (0.02%–0.05% in the diet) also inhibit specific tongue carcinogenesis in rats [24]. On the other hand, among phenolic acids, gallic acid, tannic acid and caffeic acid can in vitro cause oxidative strand breaks in DNA [8]. 

Phenolic acids are not extractable by organic solvents since they appear as insoluble covalent complexes, coupled to cell-wall polymers through ester and glycosidic links. The bound phenolic acids may be released before extraction using base hydrolysis, acid hydrolysis, or both. Many extraction procedures incorporate the application of an antioxidant as a stabilizer. Besides the conventional extraction techniques such as Soxhlet and ultrasound-assisted extractions, supercritical fluid and accelerated solvent extractions have been recently applied [105].

#### 3.3.1. Hydroxybenzoic Acids

Hydroxybenzoic acids are simple aromatic acids with strong antioxidative and anticancer activities. The most important representatives are gallic and ellagic acid abundantly present in fruits and nuts [93,106]. Additionally, being the precursors of tannins makes them even more ubiquitous although in certain aliments only. Owing to their limited distribution in food, very little is known about the absorption and metabolism of hydroxybenzoic acids [22]. Some studies report that gallic acid is extremely well absorbed, compared to other polyphenols [22]. With ingestion of 50 mg of pure gallic acid (contained in 0.8–5 L of red wine), its metabolites have reached 4 μmol/L. However, gallic acid exists in different forms in fruits, nuts, tea, and red wine, i.e., the free form, esterified to glucose (as in hydrolyzable tannins), or esterified to catechins or proanthocyanidins (as in condensed tannins) [22]. 

##### Gallic Acid

Gallic acid is one of the most widely studied and promising compounds in prostate cancer research [93]. It is predominantly present in strawberries, pineapples, bananas, lemons, red and white wines, gallnuts, sumac, witch hazel, tea, oak bark and apple peels [93,107,108]. It features several biological properties: strong anti-oxidative, anti-inflammatory, antibacterial, antiviral, anti-melanogenic, antimutagenic and anticancer activities [93,107,108]. Gallic acid exerts its biological effects by interfering with several pharmacological and biochemical pathways [107]. One of the anticancer properties is also a pro-oxidative feature that induces apoptosis in cancer cell lines [107]. Similar as in the case of catechins, this feature can also cause oxidative damage to organisms. Gallic acid has diverse effects on various tumor types at different molecular levels which make this compound an important biomolecule for therapeutic uses; it is even soluble in water [93,107,108]. Common property shared by a majority of polyphenols, including gallic acid, is selective cytotoxicity toward cancerous cells and minor or no toxicity toward normal cells [107]. Gallic acid is able to induce apoptosis by arresting cells in the S and G_1_ phase; to suppress tumor growth, cell invasion, proliferation and tumorigenesis via inhibition of angiogenesis [93]. Additionally, it modulates androgen receptor expression and lowers androgen-induced prostate-specific antigen and Fas cell surface death receptor (FAS) protein levels [93]. Therefore, gallic acid can modulate every step of carcinogenesis. Along with its established role in drug development, gallic acid is also used as an additive in food supplements to decrease the risk of cancer [107].

##### Ellagic Acid

Being a part of many species of flowering plant families, ellagic acid is an important naturally occurring hydroxybenzoic acid [106]. In plants, it is mainly present in the glucoside form or as a part of hydrolyzable tannins (glucose esters) called ellagitannins [106,108]. The richest sources of this dimeric derivative of the gallic acid include blackberries, raspberries, strawberries, cranberries, pomegranate, walnuts and pecans [93,106]. Ellagic acid possesses anti-carcinogenic, anti-oxidant, anti-inflammatory, anti-bacterial, anti-atherosclerosis, anti-hyperglycemic, anti-hypertensive, anti-fibrosis and cardioprotective effects [93,108]. Its inhibition of carcinogenesis appears to occur through a number of mechanisms [106]. It is involved in a multitude of processes that take part in angiogenesis and metastasis of several cancer types including prostate, skin, esophageal, and colon cancers [93,108]. Moreover, it induces apoptosis, reduces proliferation, inhibits invasion and motility of cancer cells [13,93,108]. Like gallic acid, ellagic acid also causes cell-specific responses, meaning that it is more reactive toward tumor cells than healthy (normal) cells [108]. Pertaining to biomarkers of tumor initiation, ellagic acid demonstrates inhibition of metabolic activation of polycyclic aromatic hydrocarbons (PAHs), nitroso compounds and aflatoxin B1, into forms that induce DNA damage [106]. The mechanism by which ellagic acid inhibits tumor initiation proceeds through the occupation of sites in DNA that might otherwise react with carcinogens or their metabolites [106]. Ellagic acid, a very stable compound, is moderately soluble in dimethylsulfoxide, slightly soluble in other organic solvents and relatively insoluble in water [106]. Owing to its potentially beneficial effects against a wide range of diseases, ellagic acids shows a great promise as a therapeutic and chemopreventive agent [108].

#### 3.3.2. Hydroxycinnamic Acids

Cinnamic acid is the precursor of hydroxycinnamic acids, a diverse group of phenolic substances possessing derivatives in almost every plant [24,93]. The most frequently encountered hydroxycinnamic acids are caffeic acid and ferulic acid [24]. Caffeic acid is, despite the name, present in many fruits such as apples, plums, tomatoes, and grapes [24]. Ferulic acid in tomatoes and beer occurs in a free form and is as such efficiently absorbed in contrast to the one in an esterified form found in grain cell walls (in the cereal) [22,24]. Intake of yet another severely important phenolic acid, chlorogenic acid, varies widely, but may be very high, up to 800 mg per day among coffee drinkers. The esterification of caffeic acid, as in chlorogenic acid, markedly reduces its absorption [22]. Being a dimeric derivative of the ferulic acid, curcumin could be also classified into this group of phenolic acids [24], however, its extremely strong biological effects caused a creation of an individual polyphenolic class named curcuminoids, presented later in this article. Notwithstanding the name, chlorogenic acid is the most abundant polyphenol in coffee (represents about 7% of the dried beans) [24]. Besides in the coffee beans, it is found in many fruits and vegetables and forms a key substrate for enzymatic oxidation that leads to browning, particularly in apples and pears [24]. 

Topically applied caffeic acid, ferulic acid, chlorogenic acid and curcumin display inhibitory effect on tumor promotion by TPA and dose-dependent reduction of tumor multiplicity [24]. Besides acting as a cancer inhibitor with antitumor and antimetastatic properties, caffeic acid also demonstrates antioxidant and antibacterial activities in vitro and possesses the capability to contribute to the prevention of atherosclerosis and other cardiovascular diseases [13,93]. Caffeic acid attenuates tumor promotion by inhibiting oxidative and inflammatory responses thereby diminishing the expression of NF-κB and COX-2 [13]. In prostate cancer cells, caffeic acid inhibits androgen receptor signaling and cell proliferation [93]. In the form of phenyl ester, proliferation proceeds even in a dose-dependent manner [93]. The compelling evidence, therefore, reveals that caffeic acid shows multiple protective effects, which can be further explored and developed towards chemoprevention, especially in prostate cancer [93].

Rosmarinic acid is a natural polyphenol antioxidant and a carboxylic acid present in many Lamiaceae herbs commonly used as culinary spices such as lemon balm, rosemary, oregano, sage, thyme and peppermint. Several studies have reported the successful use of natural phenolic antioxidants in reducing the formation of heterocyclic amines (HCAs), suspected human carcinogens formed in a meet during high-temperature grilling or cooking [109]. Such effect is possibly the result of phenolic’s superior free-radical scavenging capabilities [109]. It is demonstrated that rosmarinic acid possesses such effects as it reduces the levels of two major heterocyclic amines [109]. Moreover, the marinade containing the highest levels of rosmarinic acid together with carnosol and carnosic acid (phenolic terpenes) represents by far the strongest inhibitor of HCA formation [109]. Similarly to EGCG and genistein, rosmarinic acid proves to be a potent inhibitor of DNMT1 activity and decreases its levels in nuclear extracts from breast cancer cells, however, it is not able to demethylate and reactivate known hypermethylated genes [86].

### 3.4. Curcuminoids

Curcuminoids are polyphenols of high importance regarding their health-promoting properties as they represent 3%–5% of turmeric that has been used in traditional Indian medicine due to its antiseptic, wound healing, and anti-inflammatory properties [110]. Curcumin, a dietary yellow pigment, is the major biologically active ingredient of the plant, present also in mustards [11,24,110]. Curcumin is widely used as a food preservative and coloring agent for food, drugs and cosmetics [24]. Bioavailability of orally applied curcumin is very low, however, as proven in animals and humans, it still reaches efficacious concentrations so that it can exert beneficial health effects [111]. A plethora of evidence exists that curcumin and its derivatives possess significant anticarcinogenic activities, a majority of which is presumably attributed to its potent antioxidant capacity at neutral and acidic pH [111]. Although, it has also been assumed that its biological activities may originate from its ability to modulate epigenetic changes [11,71]. Along with quercetin, apigenin, EGCG and genistein, curcumin is able to cross the nuclear membrane enabling it to take part in epigenetic regulation [71]. Curcumin has demonstrated not only the ability to prevent epigenetic changes but even to reverse them [11,71]. Among the most important mechanisms of reversing epigenetic changes are a reduction of histone acetylation mainly via inhibition of HAT activity and inhibition of histone deacetylases that induce DNA double-stranded breaks and apoptosis [11,71]. Curcumin’s effects on epigenetic modifications are presented in detail in several articles. Other possible explanation for various capabilities of curcumin to influence several cell processes/pathways at multiple levels lies in its capacity to bind to proteins, as it has been shown to bind to at least 33 different ones [69,111]. Direct polyphenol-protein interactions may result in alternations in particular signaling transduction pathways and consequentially in modifications of higher order processes such as growth and proliferation, apoptosis, angiogenesis, metastasis and inflammation [71]. Curcuminoids have demonstrated their involvement in all of the mentioned processes, featuring their ability to suppress/influence all three stages of carcinogenesis [13]. Some of the most important anticancer activities of curcumin include diverse effects on cellular enzymes, angiogenesis and on cell adhesion [111]. 

Curcumin exhibits growth suppressive activity against a broad range of tumor types, including breast, skin, forestomach, duodenal and colon carcinogenesis [13,24,110]. Showing no significant toxic, genotoxic and teratogenic properties in phase I clinical trials, it is considered to be a safe phytochemical [71,110]. Therefore, curcumin has been subjected to further extensive clinical studies for development into a drug for treatment of various human cancers [71,110]. Most of the curcumin’s analogs have also shown very good anticancer activity in various animal models and cell lines, some even of clinical developmental importance [110]. They additionally exert anti-HIV, antimutagenic, antiangiogenic, antimalarial, anti-tubercular and anti-androgenic activities [110]. Curcuminoids definitely represent future pharmaceuticals for treatment and prevention of cancer and possibly several other currently prevailing diseases.

Conventional solid–liquid extraction, sonication, Soxhlet extraction and related techniques that have traditionally been used to extract curcuminoids comprise inevitable exposure to light, high temperatures and oxygen. Degradation of curcuminoids is a serious drawback as a consequence of the above-mentioned processing conditions. Other undesired effects are the limited efficiency of the processes and the application of the extracted curcuminoid compounds in food products. Pressurized liquid extraction represents an attractive alternative to traditional extraction processes. The technique requires smaller amounts of solvent and is even more efficient [112].

### 3.5. Stilbenes

The discovery of the stilbene class of polyphenols can be mainly attributed to the so-called “French paradox”, which revealed the connection between limited consumption of red wine (rich in antioxidants-polyphenols) and lower prevalence of many human diseases including cancers, regardless of the diet [113]. Due to its abundance in grapes and grape products, their typical representative resveratrol is one of the main targets of anticancer research [114]. Resveratrol, pterostilbene and piceatannol represent the most important constituents of stilbene class and belong to phytoalexins since they are synthesized in plants in response to fungal infection or other environmental stress [100,113,115]. Although wine may be considered a predominant bioavailable dietary source of stilbenes; grapes, various berries, peanuts and available dietary supplements are all highly relevant sources as well [83,101,114,115]. Despite their abundance in grapes and wine, resveratrol and pterostilbene are poorly bioavailable, mainly owing to their rapid metabolism and excretion [114,116]. 

Stilbenes resveratrol, pterostilbene and piceatannol possess several biological properties including strong antioxidant, anti-inflammatory and anticancer features [83,100,113]. Moreover, pterostilbene exerts analgesic activity, while resveratrol shows preventive activities towards atherosclerosis [83,100]. Preventive action of stilbenes toward various diseases can be mainly attributed to their strong antioxidant properties, anti cyclooxygenase activity and to a modulating activity of lipid and lipoprotein metabolism [83]. Notwithstanding that resveratrol is in the center of scientific attention, piceatannol shows even higher biological activity as an inhibitor of COX-2 [113]. However, resveratrol exhibits strong antiproliferative and apoptosis-inducing effects, inhibits platelet aggregation and exhibits a dose-dependent antiestrogenic activity similarly to genistein and quercetin [100,114,115,116]. Induction of apoptosis by resveratrol is also cell specific as it induces apoptosis in cells expressing wild-type p53, but not in p53-deficient cells [114]. A wide variety of tumor models demonstrates resveratrol’s abilities to inhibit initiation and promotion of progression of several cancer types including skin, breast, prostate, colon, oral, leukemia and gastric [83,100,114,115]. Piceatannol could be also used to treat various cancers as it induces an intrinsic mode of apoptosis and inhibits proliferation in various tumor cells including leukemia, lymphoma, breast, prostate, colon and melanoma [113]. Oral administration of resveratrol additionally results in a decrease in the tumor multiplicity and volume, and delays the onset of tumorigenesis [83,100]. Piceatannol also bears a simple chemical structure that is capable of interacting with a variety of receptors and enzymes and serves as an activator or inhibitor in a number of pathways [114]. Similarly, pterostilbene and piceatannol can inhibit multiple signal transduction pathways thus providing a wide variety of preventive and therapeutic options against cancer [100,113]. On the other hand, it is noteworthy that no adverse effects or toxicity have been reported for resveratrol, even at high doses [83,114]. Resveratrol’s preventive action is not limited to cancer; it shows beneficial effects on cardiovascular, inflammatory and neurological diseases along with diabetes [83]. Like other natural polyphenols, resveratrol and piceatannol are extremely photosensitive compounds with low chemical stability and consequentially, as already mentioned, limited bioavailability, which hampers their potential for development into therapeutic agents [113,115]. In particular, nanotechnological approaches could help to overcome the pharmacokinetic issues of bioactive compounds by providing improved bioavailability, overcoming the first-pass metabolism and trounce enterohepatic recirculation, by protection against degradation, enhancement in intracellular penetration and control delivery, and by reducing potential toxicity [115]. Stilbenes can be therefore used in a natural form for prevention or in their pure form for therapy, for which large doses or nanoformulation are recommended [113].

Alcohols are known as the most suitable solvents for stilbene extraction. Methanol and ethanol are most recently applied because of the suitable physical properties. Several authors have focused on studying and comparing various methods of extracting stilbenes from grape canes, including maceration at laboratory temperature, higher temperature extraction, fluidized-bed extraction, Soxhlet extraction, microwave-assisted extraction, and accelerated solvent extraction [117].

### 3.6. Tannins

Unlike the previously described classes of polyphenols, tannins are compounds of intermediate to high molecular weight consisting of oligo- and polymers of already described polyphenols [67]. Having two or three phenolic hydroxyl groups on a phenyl ring, tannins are highly hydroxylated molecules and can, therefore, interact and form insoluble complexes with carbohydrates and proteins [67,118]. The name tannin was given, consistently with such properties, to plant extracts exhibiting astringency, therefore possessing tanning capacity for transforming animal hides into leather and ability to precipitate salivary proteins [67,118,119]. The latter feature provides astringency to tannin-rich foods [67,73,118,119]. Dietary tannins can be subdivided into two major groups: hydrolyzable tannins and condensed tannins [8,68,119]. Hydrolyzable tannins are compounds containing a central core of glucose or of another polyol esterified with gallic acid, also called gallotannins with representative theogallin found in tea, or with hexahydroxydiphenic acid, also named ellagitannins after its dilactone ellagic acid with representative punicalagin present in pomegranate [8,67]. Evident from their name, these tannins can be easily hydrolyzed by acid, alkali, hot water or by enzymatic action, which yield polyhydric alcohol and phenyl carboxylic acid [67]. In some literature sources, galloylated catechins are categorized in the class of tannins instead of in the flavonoid class as is the case of this review. Condensed tannins are oligomers or polymers of flavan-3-ol or flavan-3,4-diol linked through an inter flavan carbon bond [8,67,120,121]. They are also referred to as proanthocyanidins because they are decomposed to anthocyanidins through acid-catalyzed oxidation reactions upon heating in acidic alcohol solutions [8,67,119]. Tannins yield even higher molecular weight species through intramolecular oxidation, polymerization and addition to anthocyanins [8,119]. However, in an acidic medium like wine, they also undergo cleavage reactions [8,119]. A high diversity among tannin class of substances is what probably provides their abundance in a variety of plants as food and feed [120]. However, significant errors in the quantification of the tannin content of plants exist as their solubility greatly differs [67]. In contrast to oligomeric proanthocyanidins (like proanthocyanidins B1–B8) and low-molecular-weight hydrolyzable tannins that are soluble in different aqueous and organic solvents, high-molecular-weight condensed and hydrolyzable tannins are insoluble [67]. Altogether, the main dietary sources of tannins represent food grains like sorghum, millets, barley, various beans, peas; fruits such as apples, bananas, berry fruits, dates, grapes, peaches, pears; beverages like wine and tea; nuts and cocoa beans [67,73,120,121]. 

Supercritical carbon dioxide and polar or non-polar co-solvents are most frequently used as solvents for an efficient extraction of tannins from different natural tissues. The advantages of supercritical extraction with regard to hydro solubilization and solvent extraction are low extraction temperature, short extraction time and absence of organic solvent in the extract [122].

Tannins, like many other polyphenols, exert several biological effects including antioxidative, antimicrobial, anticarcinogenic, cardiovascular system protecting, and anti-inflammatory [118,121,123]. Their strong antioxidative action reflects in the free radical scavenging activity, chelation of transition metals, inhibition of pro-oxidative enzymes and lipid peroxidation [121,123]. On the other hand, they are considered as anti-nutrients due to the formation of complexes with proteins, starch, and digestive enzymes; and due to their detrimental influence on utilization of vitamins and minerals [120,121]. Moreover, they cause browning reactions in foods, damage to the mucosal lining of the gastrointestinal tract, alteration of excretion of certain cations and increased excretion of proteins and essential amino acids [120]. Like several other polyphenols they can act as pro-oxidants catalyzing DNA degradation in the presence of transition metal ions such as copper [124]. Even though tannin components have been implicated in the high levels of cheek and oesophageal cancer in certain regions of the world, probably due to their ability to cause irritation and cellular damage rather than due to their direct action on DNA mutation, they are not necessarily the main contributing factor [121]. Additionally, they can act as co-carcinogens or promoters in inducing skin carcinogenesis [121]. Discordantly, anticarcinogenic, antimutagenic, antiproliferative activity, suppression of malignant cell migration, invasion and metastasis by tannins have been reported in vitro and in vivo [8,71,121]. 

Taking everything into account, it is not advisable to ingest large quantities of tannins, since they may possess carcinogenic and anti-nutritional activities, thereby posing a risk of adverse health effects [121]. However, in small quantities, they may be beneficial to human health and if not themselves, surely some of their constituting substances may contribute to health-promoting activities [121]. Many issues considering tannins still remain to be resolved as they are not so thoroughly examined compared to simpler polyphenols. 

### 3.7. Lignans

Lignans are dimeric compounds that contain a 2,3-dibenzylbutane structure formed by the dimerization of two coniferyl or sinapyl alcohol units (derivatives of cinnamic acid) [93,96]. Lignans are the most ubiquitous phytoestrogens because they exist as minor constituents of many plants, where they are involved in plant cell wall formation [70]. Predominant sources of lignans, especially secoisolariciresinol and matairesinol, are flaxseed meal and flour [93]; additionally, they can be found in varying concentrations in soybeans, whole grains, fruits and vegetables [70]. In extra virgin olive oil, the fraction of lignans is approximately twice that of total simple phenols, with lignans (+)-1-acetoxypinoresinol and (+)-pinoresinol being the major components of the phenolic fraction [125,126]. Most lignans appear to pass through the intestinal tract as fibers [93]. On the other hand, dietary secoisolariciresinol and matairesinol can be converted by intestinal microflora to enterodiol and enterolactone, major lignans identified in humans, which are absorbed through enterohepatic circulation [93,126].

Lignans are known for their high antioxidant activity, the strongest among phenolic components of the olive oil, and for inhibiting lipid peroxidation [125,126]. Moreover, partially defatted flaxseed (containing lignans and flaxseed gum) lowers LDL cholesterol by approximately 8%, however, the pro-oxidant activity of such flaxseed has also been demonstrated [70]. Similarly to isoflavones, lignans possess both estrogenic and antiestrogenic activities [93,126]. These properties might play an important role in lignans’ inhibition of skin, breast, colon and lung cancer cell growth [125]. Like in the case of genistein, early life exposure to lignans apparently affects the mammary gland development, which causes the reduction of tumorigenesis [93,126]. Representing the major fraction of olive oil phenolics, lignans could significantly contribute to the health-promoting effects of the Mediterranean diet [126]. Evidence suggests that phytoestrogens like isoflavones and lignans can provide protection against a wide range of clinical conditions: already mentioned cancers, cardiovascular diseases, menopausal symptoms and osteoporosis [70]. Still, caution regarding time, dose and manner of administration is needed as improper estrogenic activity can result in negative side effects, especially in premenopausal women and infants [93].

The extraction methods comprise the removal of the lignan glycosides from the plant matrix with an alcoholic solvent system. In the followed step, acid hydrolysis to release the aglycons is performed. A reversed-phase high-performance liquid chromatography with diode array detection system may be used for initial separation and detection of the lignans. Lignan trimethylsilyl ether derivatives are detached by gas chromatography/mass spectrometry [127]. In the following Table 1, a summation of described biological activities and applied extraction methods of the main polyphenol representatives belonging to various classes, is made.

### 3.8. Synergistic Effects of Combinations of Polyphenols and Their Combinations with Drugs/Therapies

In Section 3, a review on the biological effects of specific polyphenols has been presented, however, we must not overlook the power of various combinations of polyphenols or their combinations with other phytochemicals, pharmaceuticals and therapies. A plethora of evidence clearly reveals that combinations of several active substances have enormous advantages over individual substances. Such combinations not only possess all the activities of individual compounds but also provide synergistic enhanced beneficial effects and prevent potential toxicity, as lower concentrations of each of the consisting compounds are needed for achieving therapeutic effects. Many health-promoting effects of polyphenols are elucidated from epidemiological studies showing an inversed connection between consummation of certain fruits or vegetables and cancer risk, thus some beneficial effects may not even originate from individual polyphenols, but rather from combinations of phytochemicals (a majority representing polyphenols) present in these aliments. 

The role of polyphenols as secondary plant products is to provide protection to plants against several threats such as microbes, UV radiation or predators. Evolution has afforded them with a variety of strategies and multiple fronts of attack to accomplish these functions, rather than single compounds to which a pathogen or predator could become resistant [101]. As our diet mainly consists of plants and their products; this multiplicity in bioactive phytochemicals brings us benefits of the interaction of various polyphenols with their therapeutic targets [101]. Such interactions can be: (a) positive, named potentiating interactions; (b) inhibitory or (c) enhancing, where concomitant compounds which are not themselves bioactive, work together with a bioflavonoid to enhance its bioavailability or absorption [101]. Even though several polyphenols exert significant anticancer activities on their own, as already mentioned, the problems considering their bioavailability, fast metabolism and excretion are inevitable, affecting the dose reaching target cells [18]. One possible solution to this issues is also combining polyphenols, or polyphenols and other anti-cancer drugs/therapies to achieve the same or even enhanced antitumor effects using lower doses of an individual polyphenol/drug [18,19]. By doing so, possible high dose side effects, drug resistance or even toxicity of polyphenols, drugs and therapies, is prevented and even reduced in the case of existing ones [18,19]. Copious studies have shown that treatment with a combination of polyphenols is more effective in inhibiting cancer formation and growth than treatment with a single polyphenol [18,19,71,101]. It should be noted that some plant extracts, as a mixture of various phenolic and non-phenolic compounds, display anticancer proprieties, however, these properties cannot be strictly assigned to any particular compound, but rather represent the consequence of their synergistic effects [19,71]. One can find numerous reports stressing that the interaction between these components is crucial for beneficial health effects of diverse phytochemicals (polyphenols) in reducing the risk of various degenerative diseases [19,71].

Synergistic action of flavonoids (mostly anthocyanins) is also responsible for the antiplatelet activity of red wine and grape juice, which without strong interactions between components of grape skin and grape seed would not be possible [101]. Several studies have been performed regarding the synergistic effects of the most potent anticarcinogenic polyphenols like EGCG, curcumin, resveratrol, quercetin and genistein with other less potent anticarcinogenic polyphenols, polyphenols that on their own show no activity and with established drugs/therapies [18]. Such interactions increase therapeutic effects by blocking one or more targets of the signal transduction pathways [19]. One of such studies even demonstrates that combination of EGCG and curcumin allows for up to 8-fold dose reduction of EGCG in the mixture for the same therapeutic effect [19].

#### Whole Extracts from Various Plant Sources vs. Isolated Polyphenols

Countless studies have revealed antioxidative and anticancer activities of polyphenols contained in tea extracts or tea extracts as a whole, especially for green and black tea. Synergy is what makes a significant difference in biological effects between tea as a whole and isolated specific tea catechins (like the most potent EGCG). Similar effects are observed with other extracts, polyphenol fraction comparing to the whole, such as fruits and fruit juices, especially the ones made of grapes, berries, citrus fruits, pomegranate, apple or peach. Moreover, some beverages like red wine, beer, coffee, cocoa and other cocoa products possess strong anti-oxidative and anticancer activities. Of course, certain grains or cereal like flaxseed and most importantly vegetable and seed oils, predominantly olive oil, exert favorable effects on human health as well.

One should consider that well balanced raw (unprocessed and organic) foods in normal quantities are always beneficial, whereas a growing number of processed foods consisting of healthy aliments, like fruits, vegetables and grains, can regardless of the dose be deleterious to our health, or even carcinogenic, mostly due to processing, pesticides or inappropriate additives. 

Taking everything into consideration, polyphenols are important substances of plant-based part of our diet and as such, they serve to protect us from numerous threats like microbes, radiation, several toxins and of course various diseases. An appropriate diet, high in diverse polyphenols, is even capable of treating some already developed diseases including cardiovascular disorders and cancer. Moreover, in combination with prescribed therapies it enhances their action and reduces or prevents possible side effects and toxicity.

## 4. Conclusions and Future Perspectives

Polyphenols represent a large and diverse group of secondary plant metabolites, therefore they are abundantly present in a majority of fruits and vegetables, even in larger quantities than vitamins. Possessing several biological effects, they ought to represent important human nutrients. Distinct features of polyphenols originate from their role to defend plants against reactive oxygen and nitrogen species, UV light, pathogens, parasites and predators. Their biological activities can provide us with effective protection or even cure for several prevailing diseases, especially various types of cancer. Omnipresence, specificity of the response and absence or low toxicity are crucial advantages of polyphenols as anticancer agents. 

Agricultural residues possess a great potential as a source of antioxidants, many of which belong to polyphenols. Extraction of polyphenols from the agricultural wastes seems a suitable technique for the isolation of these highly valuable, thermolabile compounds. However, extraction of polyphenols is hindered by two main factors: firstly, polyphenols may appear in plant tissues complexed with sugars, proteins or they may create polymerized derivatives with an increased resistance against an effective isolation which mainly depends on proper solvent selection in addition to terms and conditions of the extraction. Secondly, polyphenols are susceptible to oxidation. The recovery of phenolic compounds from plant materials is influenced by the extraction technique, the extraction time and temperature, the solvents used, the solvent to solid ratio and by the intensity of waves. High temperature, long extraction times and alkaline environment cause their degradation. Antioxidant power is usually related to the phenolic content and solvent extracting power represents the most important factor affecting antioxidant capacity. Compared to alternative methods, microwave-assisted and ultrasonication extraction techniques are considered as much more promising and have shown a greater potential and better efficiency for the extraction of polyphenols with a high antioxidant activity. The stability of proanthocyanidins can then be maintained for over two weeks by storing the extract in a freezer. A possible solution to avoid the degradation of polyphenols lies in encapsulation which also represents the most common solution in relation to their poor bioavailability that significantly reduces the polyphenol dose reaching the target cells. The encapsulation features additional advantages of masking the flavor, of control and targeted release. An alternative solution is the use of combinations of various polyphenols which brings synergistic effects resulting in lowering of the required therapeutic dose of a particular polyphenol. The combination of polyphenols with existing drugs and therapies has also shown promising results and has importantly reduced their toxicity. A healthy lifestyle remains crucial for disease prevention; the most important part represents a balanced diet abundant with various fruits and vegetables that contain vital mixtures of polyphenols. 

Several encapsulation techniques (spray drying, spray chilling and cooling, coacervation, fluidized bed coating, liposome entrapment, rotational suspension separation, extrusion and inclusion complexation) have in the past years been used to convert liquid components into solid particles and to provide a means for their controlled release [128]. Techniques applying supercritical fluids [129,130,131] have demonstrated to successfully increase the stability of bioactive compounds or pharmaceuticals and their utilization can be certainly forecasted to grow [129]. As already mentioned, polyphenols have recently attracted a great interest of the functional foods, nutraceutical and pharmaceutical industries due to their potential health benefits to humans. Recent research is focused on the fortification of foods with active compounds as a mean of improving their nutritional value without compromising the organoleptic properties. Microencapsulation of anthocyanins with different biopolymers through spray drying is a subsequent step to be coupled with a suitable extraction technique in order to obtain products with improved physical and chemical properties (porosity, stability, solubility and dispersibility) compared to the original material allowing for easier handling, storage and transportation [132].

**Figure 4:**
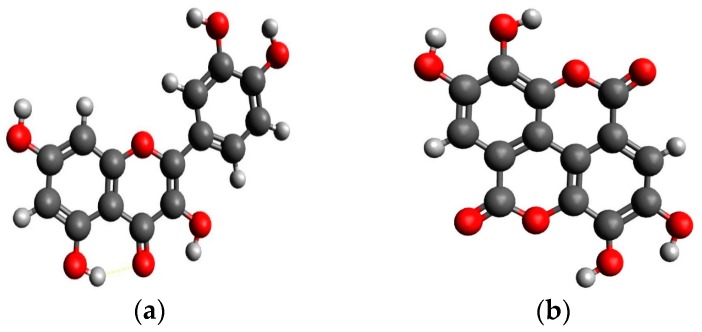
Quantum chemical models of (**a**) quercetin and (**b**) ellagic acid. Carbon atoms are depicted in gray, oxygen in red and hydrogen in white.

Scientists have studied the action of polyphenols through biological processes in various cell cultures, laboratory animals and using epidemiological studies. However, not many have focused on the cancer preventive action of polyphenols from the mechanistic point of view. Meaning, how they interact with carcinogenic or other toxic substances. Employing molecular modeling and computer simulations enables exploring these questions without exposing oneself to carcinogenic substances [133,134,135,136,137]. It can provide answers like which polyphenol is more reactive toward specific carcinogenic/toxic substance and thus more able to potentially prevent mutagenic/genotoxic DNA damage and consequently also the formation of cancer and other diseases. It would be therefore important to consider investigating polyphenols also from the computational point of view in the future. Computational models like the ones depicted in Figure 4, obtained using quantum chemical simulations, can be used to determine the scavenging potential of natural polyphenols towards various chemical carcinogens by exploring the corresponding kinetics in a safe and environmentally-friendly manner. In addition, such simulations can also direct the selection of the best extraction medium via solvation free energy calculations.

## Figures and Tables

**Table 1:** Polyphenol classes, their typical representatives, main sources, applied extraction methods and described biological effects.

Polyphenol	Sources	Extraction	Effects	References
Epigallocatechin 3-O-gallate (EGCG) (flavonoids/catechins)	Tea, fruits (apples, grapes, berries), red wine, chocolate	Maceration, ultrasonic extraction, microwave extraction, stirring	Antioxidative, pro-oxidative, pro-apoptotic, anti-proliferative, suppression of growth and invasion, antiangiogenic, antimetastatic, antimutagenic, anti-inflammatory, inhibition of telomerase activity and lipid peroxidation, modulation of estrogen activity, modulation and reversal of epigenetic changes	[1,8,10,13,17,18,35,69,81,82,84,85,86]
Apigenin (flavonoids/flavones)	Aromatic plants (chamomile, parsley, oregano, thyme), grapefruit, oranges, onions	Organic solvent extraction using methanol, ethanol and propanol as well as their mixtures	Antioxidative, anti-mutagenic, anti-inflammatory, anti-viral, inhibition of tumor growth, pro-apoptotic, suppression of tumor progression, anti-invasive, antiangiogenic, antimetastatic, anti-proliferative, modulation of epigenetic changes	[8,13,36,93,94]
Quercetin (flavonoids/flavonols)	Vegetables (onions, broccoli), fruits (apples, apricots, berries), nuts, seeds, tea, wine, cocoa	Subcritical water extraction, ultrasonic-assisted extraction	Strongly antioxidative; pro-oxidative, antiviral, inhibition of tumor formation and migration, pro-apoptotic, anti-proliferative, antimetastatic, anti-angiogenic, inhibition of lipid peroxidation, reduction of tumor incidence and multiplicity, prevention of GJIC inhibition, modulation of epigenetic changes	[5,8,10,11,13,24,35,70,71,81,82,86,91,95,96,97]
Fisetin (flavonoids/flavonols)	Strawberries, apples, persimmons, grapes, onions and cucumbers	HCl, EDTA, and formic acid extraction methods	Antioxidative, pro-apoptotic, induction of cell cycle arrest, inhibition of androgen signaling and tumor growth, antiproliferative, decrease in viability of tumor cells	[8,22,35,36,37,91,93]
Naringin (flavonoids/flavanones)	Citrus fruits, tomatoes, aromatic plants	Supercritical fluid extraction, conventional soxhlet extraction with different volatile solvents	Antioxidative, anti-inflammatory, anti-metastatic, delayed tumor development, reduction of tumor incidence, blocking of peroxide cytotoxicity and apoptosis of healthy cells	[8,31,32,33,69,82,91,94]
Naringenin (flavonoids/flavanones)	Citrus fruits, tomatoes, aromatic plants	Supercritical fluid extraction, methanol extraction	Antioxidative, anti-metastatic, antiproliferative, stimulation of DNA repair after oxidative damage	[7,31,32,33,91,92,94,98]
Hesperetin (flavonoids/flavanones)	Citrus fruits, tomatoes, aromatic plants	Microwave-assisted extraction, extraction with organic solvents and mixtures (DMSO–methanol)	Antioxidative, inhibition of malignancy, antimetastatic, antiviral, anti-inflammatory	[31,32,33,70,83,91,94,97]
Genistein (flavonoids/isoflavones)	Legumes, especially soya	Sub- and supercritical fluid extraction (pressurized hot water extraction, carbon dioxide)	Antioxidative, anti-invasive, anti-inflammatory, anti-metastatic, delay/repression of tumor developement/growth, reduction of tumor multiplicity and volume, pro-apoptotic, antiproliferative, estrogenic activity, prevention of GJIC inhibition, modulation of epigenetic changes	[1,3,8,10,11,17,22,24,31,70,71,81,86,88,100]
Xanthohumol (flavonoids/chalcones)	Hops, beer	Supercritical fluid extraction	Antioxidative, anti-inflammatory, antiestrogenic, modulation of enzymatic action, pro-apoptotic, anti-invasive, suppression of tumor growth, anti-proliferative, targeting several processes	[6,32,76,100,102,103,104]
Isoliquiritigenin (flavonoids/chalcones)	Licorice, shallot and bean sprouts	Soxhlet extraction, supercritical fluid extraction	Potent antioxidant, anti-inflammatory, antimetastatic, anti-invasive, anti-adhesive, inhibition of migration	[100,122]
Gallic acid (phenolic acids/hydroxybenzoic acids)	Berries, pineapples, bananas, lemons, wines	Soxhlet extraction, ultrasonic-assisted extraction, microwave-assisted extraction	Antioxidative, pro-oxidative, anti-inflammatory, antibacterial, antiviral, anti-melanogenic, antimutagenic, suppression of tumor growth, anti-invasive, antiproliferative, inhibition of tumorigenesis, anti-angiogenic, modulation of androgen receptor	[8,22,81,93,105,106,107,108]
Ellagic acid (phenolic acids/hydroxybenzoic acids)	Berries, pomegranate, walnuts and pecans	Ultrasound-assisted extraction	Antioxidant, anti-inflammatory, anti-bacterial, anti-angiogenic, antimetastatic, pro-apoptotic, anti-proliferative, anti-invasive, inhibition of motility	[13,93,105,106,108]
Rosmarinic acid (phenolic acids/hydroxycinnamic acids)	Herbs from the Lamiaceae family	Extraction with organic solvents, supercritical fluid extraction	Antioxidative, reduction of HCA formation, modulation of epigenetic changes	[86,105,109]
Curcumin (curcuminoids)	Turmeric, mustard	Extraction with different solvents in pure form or their mixtures	Antioxidative, anti-angiogenic, anti-adhesive, tumor growth suppressive, antiproliferative, proapoptotic, antimetastatic, anti-inflammatory, modulation and reversal of epigenetic changes	[11,13,24,71,110,111,112]
Resveratrol (stilbenes)	Red wine, grapes, berries, peanuts	Supercritical fluid extraction, pressurized liquid extraction (water, methanol and other organic solvents)	Antioxidative, anti-inflammatory, anti-cyclooxygenase, antiproliferative, proapoptotic, antiestrogenic, modulation of lipid metabolism, inhibition of platelet aggregation	[83,100,113,114,115,116,117]
Pterostilbene (stilbenes)	Red wine, grapes, berries, peanuts	Maceration, extraction at elevated temperature, fluidized-bed extraction, Soxhlet extraction, microwave-assisted extraction and accelerated solvent extraction	Antioxidative, anti-inflammatory, analgesic, anti-cyclooxygenase, pro-apoptotic, antiproliferative, modulation of lipid metabolism	[83,100,113,114,115,117]
Piceatannol (stilbenes)	Red wine, grapes, berries, peanuts	Maceration, extraction at elevated temperature, fluidized-bed extraction, Soxhlet extraction, microwave-assisted extraction and accelerated solvent extraction	Antioxidative, anti-inflammatory, anti-cyclooxygenase, modulation of lipid metabolism	[83,100,113,114,115,117]

## Abbreviations

The following abbreviations are used in this manuscript:

ABTS	2,2′-azino-bis(3-ethylbenzothiazoline-6-sulphonic acid)
Akt	protein kinase B
AP-1	activator protein 1
AR	androgen receptor
Bax	Bcl-2 associated X protein
Bcl-2	B-cell lymphoma 2
C/EBPβ	CCAAT/enhancer-binding protein beta
COX	cyclooxygenase
DNMT	DNA methyltransferase
DPPH	2,2-diphenyl-1-picrylhydrazyl
EC	epicatechin
ECG	epicatechin-3-gallate
EGC	epigallocatechin
EGF	epidermal growth factor
EGFR	epidermal growth factor receptor
EGCG	epigallocatechin-3-gallate
ER	estrogen receptor
FAS	Fas cell surface death receptor
GC	gas chromatography
GJIC	gap junction intracellular communication
HAT	histone acetyltransferase
HCA	heterocyclic amine
HER2	human epidermal growth factor receptor 2
HPLC	high-performance liquid chromatography
IGF	insulin-like growth factor
IL-1RI	interleukin 1 receptor type I
iNOS	inducible nitric oxide synthases
JAK	Janus kinase
LDL	low-density lipoprotein
LOX	lysyl oxidase
MAPK	mitogen-activated protein kinases
MGMT	O^6^-methylguanine DNA methyltransferase
miRNA	micro RNA
MMP	matrix metalloproteinase
MLH1	MutL homolog 1
MS	mass spectrometry
NF-κB	nuclear factor kappa B
NO	nitric oxide
PAH	polycyclic aromatic hydrocarbons
PI3K	phosphatidylinositol-3-kinases
RAR	retinoic acid receptor
ROS	reactive oxygen species
SCF	supercritical fluids
STAT	Signal Transducer and Activator of Transcription
TGF	transforming growth factor
TPA	tetradecanoyl phorbol acetate
VEGF	vascular endothelial growth factor
Wnt	wingless-related integration site
